# Phosphorylated lantibiotics-producing commensals integrate into the human oral microbiome to suppress pathogens and promote microbiome homeostasis

**DOI:** 10.1038/s41522-026-00976-y

**Published:** 2026-04-03

**Authors:** Abdelahhad Barbour, Yehoshua Bendayan, Cara Marks, Yan Hei Kelly Choi, Morvarid Oveisi, Mitchell Callaghan, Chunxiang Sun, Sina Zargaran, Max Xia, Dempsey Wood, Leif Smith, Jeffrey S. McLean, Tony Mazzulli, Michael Glogauer

**Affiliations:** 1 Faculty of Dentistry, University of Toronto, Toronto, ON Canada; 2 Ostia Sciences Inc, Toronto, ON Canada; 3 Department of Biology, Texas A&M University, College Station, TX USA; 4 Department of Periodontics, School of Dentistry, University of Washington, Seattle, WA USA; 5 Department of Microbiology, Sinai Health System/University Health Network, Toronto, ON Canada; 6 Department of Laboratory Medicine and Pathobiology, University of Toronto, Toronto, ON Canada; 7 Department of Dental Oncology, Princess Margaret Cancer Centre, Toronto, ON Canada

**Keywords:** Diseases, Microbiology

## Abstract

Commensal bacteria produce antimicrobial peptides (AMPs) to maintain microbiome homeostasis, yet the traits underlying this resilience and their translation into biotherapeutics remain understudied. Phosphorylated lantibiotics (pLANs) are a recently identified class of ribosomally synthesized and post-translationally modified peptides (RiPPs), with dual antimicrobial and pro-immune activities. In this manuscript, we explore the potential of commensals’ pLANs biosynthesis as a mechanism for pathogen suppression and microbiome homeostasis. Subgingival metagenomics revealed that oral health correlates with *Streptococcus salivarius* enrichment and an increased prevalence of streptococcal RiPP biosynthetic gene clusters. Guided by these associations, we screened 80 *S. salivarius* isolates, identifying a small subset producing pLANs with potent activity against *Porphyromonas gingivalis*, vancomycin-resistant *Enterococcus faecium*, and multidrug-resistant *Streptococcus pneumoniae*. A representative lead strain, SALI-10, exhibited robust epithelial adhesion and a sorbitol-driven metabolic adaptation that enhances pLANs expression. In human-derived dysbiotic biofilms, SALI-10 stably engrafted, suppressed periopathogens, reduced antibiotic-resistance genes, and enriched acid-buffering pathways. In a first-in-human feasibility trial, daily oral administration of SALI-10 for one week yielded increased pLANs signals, pathogen depletion, and reduced oral neutrophil counts. Ultimately, pLANs-producing *S. salivarius* acts as a precision commensal to restore ecological balance, defining a mechanistically grounded and microbiota-mediated strategy to prevent oral and respiratory infections.

## Introduction

Oral and respiratory infections rank among the most common and recurrent diseases worldwide^1^. They affect billions worldwide and often require antibiotics, which can disrupt the ecological balance of human microbiomes. Strains of multidrug-resistant (MDR) bacteria, such as *Streptococcus pneumonia* and *Streptococcus pyogenes*, inhabit the oral and oropharyngeal areas^2–4^. Additionally, periodontal pathogens like *Porphyromonas gingivali* are associated not only with chronic oral inflammatory diseases but also with systemic health issues, including atherosclerosis^5^ and neurodegenerative disorders^6,7^. These risks are exacerbated by the limited specificity of traditional antimicrobials, which indiscriminately eliminate beneficial organisms and accelerate the spread of resistance genes^8^. This imbalance promotes the emergence and spread of antibiotic resistance, highlighting the urgent need for precision-based, microbiota-informed alternatives to conventional antimicrobial therapy^9–11^.

Commensal microbes residing in the oral cavity play a crucial role in shaping microbial community structure and influencing host immune responses. However, the factors determining which taxa can successfully persist, outcompete pathogens, and modulate inflammation remain poorly explored. Recent studies suggest that niche-adapted keystone species can disproportionately influence microbiome function, particularly through their production of antimicrobial metabolites and coordinated colonization strategies^12–14^.

*Streptococcus salivarius*, a key oral colonizer, supports oral microbial balance by buffering pH (through urease and arginine deiminase) and adhering to mucosal surfaces^15^. Certain strains of *S. salivarius* produce lantibiotics called salivaricins, AMPs that inhibit competing pathogens^16^. These lantibiotics are typically non-phosphorylated RiPPs, as their biosynthesis involves dehydration, phosphorylation, and subsequent phosphate elimination^17^. Until recently, no naturally occurring lantibiotics with stable phosphorylation had been described.

**Fig. 1:**
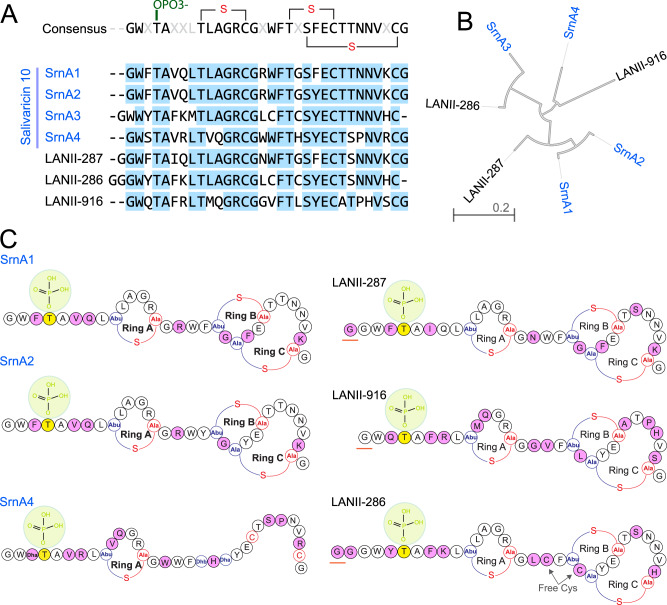
Structural analysis of the pLANs family. **A** Amino acid sequence alignment of natural salivaricin 10 core peptides produced by *S. salivarius* SALI-10 and related variants discovered via genome mining. Annotated macrocycle locations are indicated, with the N-terminal phosphate group (OPO3-) highlighted in green. Conserved amino acids are marked in blue. **B** A hierarchical clustering diagram illustrates the relationship between natural salivaricin 10 lantibiotics and other recombinant streptococcal salivaricin 10 variants identified through genome mining. Branch length on the tree scale indicates amino acid substitutions per position. **C** Structural comparison between salivaricin 10 pLANs (SrnA1, SrnA2, and SrnA4) and recombinant variants (LANII-287, LANII-916, and LANII-286). The N-terminal phosphate group is highlighted in green, denoting the phosphorylation location at the N-terminal threonine, shown in yellow. Amino acid residues highlighted in magenta in this pLANs group may be non-conserved. In the recombinant lantibiotics (LANII-287, LANII-916, and LANII-286), underlined residues represent N-terminal glycine retained after protease cleavage and are not part of the natural core sequence.

Our group has previously identified a rare subset of *S. salivarius* that produces novel lantibiotics featuring a key phosphothreonine residue, which is essential for bioactivity. We term these pLANs “salivaricin 10,” which is known for its selective antimicrobial properties. Unlike conventional lantibiotics, pLANs retain phosphate during maturation, which is essential for their unique immunomodulatory activities^18^. Salivaricin 10 targets MDR pathogens associated with oral and respiratory infections while sparing the beneficial microbiota^18^. Genome mining of human microbiome data identified new variants of salivaricin 10 from an oral *Streptococcus* genome. Cloning and expression confirmed a phosphothreonine residue at the same position as in salivaricin 10 peptides^19^. To date, six pLANs have been identified from the human oral microbiome: three peptides (SrnA1, SrnA2, SrnA4) from the natural producer strain *S. salivarius* SALI-10 and another three (LANII-286, LANII-287, LANII-916) sourced from metagenomics data and studied through cloning and structural analysis (Fig. 1).

*S. salivarius* strain SALI-10 has emerged as a model for integrating antimicrobial production with metabolic and ecological adaptation. In the current manuscript, we show that pLANs-producing strains, including SALI-10, can ferment sorbitol, a sugar alcohol commonly found in oral care products but not widely metabolized by oral microbes^20^. We found that in SALI-10, sorbitol fermentation is associated with the upregulation of pLANs biosynthesis, bypassing catabolite repression that limits lantibiotic production under nutrient-rich conditions^18,21,22^. SALI-10 exhibits unique strain-specific adhesins that bind to oral surfaces. It maintains pLANs expression in complex microbial environments, suppressing pathogenic species, diminishing virulence genes, and enhancing microbiome resilience.

To evaluate the clinical potential of this discovery, we conducted a pilot human intervention study in which SALI-10 cells, which produce pLANs, were transplanted into the oral cavity of healthy subjects. This bacterial transplantation resulted in successful colonization of the pLANs-producing strain, reduced oral polymorphonuclear neutrophils (oPMN) count, and decreased pathogenic taxa, consistent with the mechanistic role of pLANs in modulating microbial and immune dynamics.

This work presents a refined model of commensal-mediated ecological defense, laying the groundwork for a translational probiotic/biotherapeutic platform that integrates metabolic regulation, host colonization, and immune modulation. It advances the broader goal of harnessing microbiota-derived molecules for therapeutic benefit in oral and respiratory health.

## Results

### *S. salivarius* and the biosynthetic potential of the oral microbiome are associated with health and show a negative correlation with pathogens

**Fig. 2:**
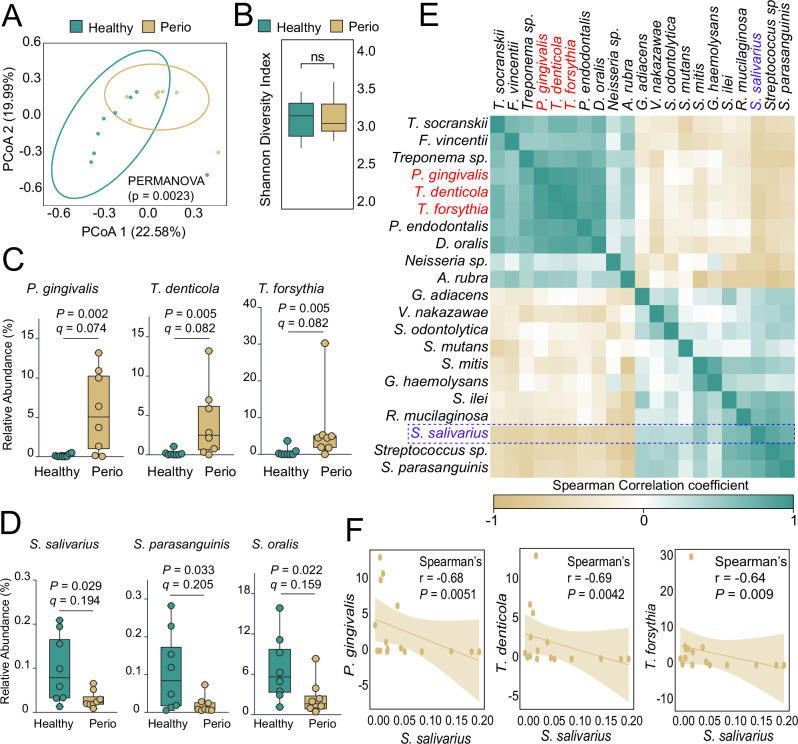
*S. salivarius* is a major contributor to health and is negatively correlated with PD. **A** Principal coordinate analysis plot analysis displaying significant composition shift of bacterial communities in the subgingival biofilm of PD patients compared to healthy subjects. **B** Shannon’s diversity index shows no significant differences in bacterial community diversity between PD patients and healthy subjects. **C** Box plots showing the relative abundance of the red complex pathogens in the subgingival biofilm of PD patients compared to healthy subjects. **D** Box plots showing the relative abundance of the streptococcal species in the subgingival biofilm of PD patients compared to healthy subjects. **E** Spearman correlation matrix by species abundance showing *S. salivarius* as the major microbial contributor. **F** *S salivarius* negatively correlated with *P. gingivalis*, *T. denticola*, and *T. forsythia*.

To identify key commensal species negatively correlated with oral infections, we performed shotgun metagenomics on subgingival samples from both healthy individuals and patients with periodontitis (PD) (see Table S1 for demographic details). Principal coordinate analysis using Bray-Curtis distances revealed a significant shift (PERMANOVA, *P* < 0.05) in the bacterial communities between healthy controls and PD patients (Fig. 2A). However, the two groups had no significant changes in the bacterial alpha diversity (Shannon diversity index, *P* > 0.05) (Fig. 2B). Microbiome Multivariable Association with Linear Models (MaAsLin2) analysis, including age as a covariate, was used to model associations between relative bacterial abundance and disease status, with several taxa showed age-associated effects (Fig. S1). The relative abundance of PD-associated “red complex” (*P. gingivalis*, *Tannerella forsythia*, and *Treponema denticola*) was significantly higher in subgingival biofilms of PD patients compared to healthy controls, where these pathogens were almost undetected in health (Fig. 2C).

Despite the small cohort size, these patterns align with the oral biofilm dysbiosis in PD, especially concerning the red complex^23^. On the other hand, oral commensal streptococci, including *S. salivarius*, *S. mitis, and S. oralis*, were at significantly higher levels in healthy subjects compared to PD patients (Fig. 2D). Spearman’s rank correlation coefficient analysis revealed that *S. salivarius* is the major microbial contributor within the microbial communities that correlates to health (Fig. 2E). Conversely, *S. salivarius* showed a positive correlation with oral commensals, including oral streptococci (Fig. 2E). In contrast, *S. salivarius* demonstrated negative correlations with the red complex bacteria, including *P. gingivalis* (Spearman’s *r* = −0.68, *P* < 0.01), *T. denticola* (Spearman’s *r* = -0.69, *P* < 0.01), and *T. forsythia* (Spearman’s *r* = −0.64, *P* < 0.01) (Fig. 2F).

**Fig. 3:**
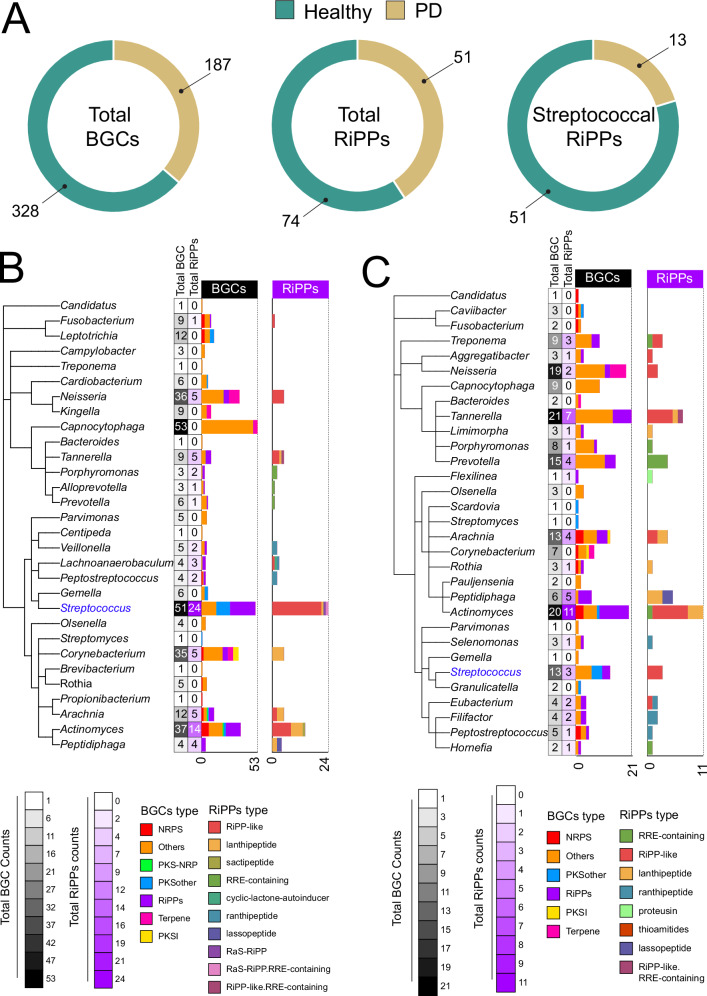
Prevalence and diversity of biosynthetic gene clusters in the oral microbiome during health and disease. Assembled metagenomes from the oral microbiota were analyzed using AntiSMASH for BGC mining, followed by the Big-escape pipeline (methods). **A** An overview of the secondary metabolite biosynthetic capacity in healthy versus PD oral metagenomes, highlighting greater biosynthetic potential in healthy samples. Additionally, total RiPPs and Streptococcal RiPPs are more abundant in the healthy oral microbiome compared to PD. **B** Elevated total BGC counts in certain genera, including *Streptococcus*, *Capnocytophaga*, *Actinomyces*, *Neisseria*, and *Corynebacterium*, characterize the biosynthetic potential of the oral microbiome in health. Notably, the genus *Streptococcus* showed the highest number of RiPPs. **C** The biosynthetic potential of the oral microbiome in PD reveals higher total BGCs counts in certain disease-associated genera such as *Tannerella*, *Treponema*, *Porphyromonas*, and *Prevotella*. Conversely, streptococcal BGCs and overall RiPPs counts are lower in PD patients compared to healthy individuals.

Given previous reports indicating that host-derived microbes can produce a variety of bioactive secondary metabolites that may influence the human microbiome^19^, we aimed to investigate the biosynthetic potential of oral microbiome samples from both healthy individuals and those with PD. Our findings reveal that healthy subjects’ subgingival species exhibit a significantly higher prevalence of biosynthetic gene clusters (BGCs) compared to patients with PD (Fig. 3). In particular, we observed significantly greater abundances of streptococcal RiPPs in healthy subjects compared to those with disease (Fig. 3). These findings suggest that enhancing the oral microbiome’s *S. salivarius* and the streptococcal biosynthetic capacity may help maintain health and combat disease-associated microbiota.

### pLANs in *S. salivarius* have relatively lower prevalence but exhibit superior antimicrobial activity compared to AMPs produced by other *S. salivarius* strains

Following the clinical outcomes observed above, we sought to identify advantageous *S. salivarius* isolates from healthy individuals with considerable biosynthetic potential for therapeutic development. To achieve this, we assembled a collection of *S. salivarius* strains derived from the oral microbiomes of healthy subjects (*n* = 80). We screened for the secretion of AMPs by *S. salivarius* to ensure the isolation and identification of strains with significant biosynthetic potential. Since earlier reports indicated that little to no lantibiotic salivaricins are produced in liquid cultures, we cultivated the isolates on rich agar medium. Peptide extracts were then prepared by freezing and thawing the agar cultures. The resulting liquid was extracted with chloroform and purified using C18 solid-phase extraction, as described previously^18^ (Fig. S2A). All bacterial cultures were processed using identical production volumes, and downstream peptide extraction and purification were performed in parallel (Supplementary Materials). Final peptide fractions were normalized to 1 mg/mL prior to antimicrobial testing. These normalized extracts were then assessed for antimicrobial activity against *P. gingivalis*, a MDR strain of *S. pneumoniae*, and vancomycin-resistant *Enterococcus faecium* (VRE) using a well diffusion assay (Supplementary Materials). Only four strains (5% of the isolates) produced AMPs effective against all three pathogens. Twelve strains (15%) produced AMPs that inhibited *S. pneumoniae* and *E. faecium*, while another twelve strains (15%) inhibited only *S. pneumoniae*. Notably, over half of the isolates (65%) did not produce any AMPs effective against the targeted pathogens (Fig. S2B), indicating that the antimicrobial potential is determined more by strain-specific biosynthetic capability than by species identity alone. The AMPs from strains SALI-10, SALI-11, SALI-64, and SALI-67 exhibited significant antibacterial activity. PCR analysis confirmed the presence of the salivaricin 10 BGC in these strains (Table S2), and mass spectrometry validated the expression of salivaricin 10 consisting of pLANs SrnA1, SrnA2, and SrnA4 by these strains (Fig. S2C). Recognizing that pLANs can boost neutrophil phagocytosis against pathogens due to their phosphorylated structure^18^, we examined AMPs from various *S. salivarius* strains for their capacity to enhance this pro-immune activity in vitro. Our findings indicated that only pLANs, and not other AMPs from *S. salivarius*, could enhance neutrophil phagocytosis of pathogenic bacteria (Fig. S2D, E). Importantly, this screening and extraction pipeline was not selective only for pLANs. The same workflow successfully isolated other non-pLANs, including salivaricin A2^24^ and salivaricin B^25^, from control *S. salivarius* strains (Fig. S3), validating the robustness of the production, extraction, and detection approach across distinct lantibiotic classes. These data highlight that while pLANs-producing *S. salivarius* strains have relatively lower prevalence in the human oral cavity, they possess substantial antimicrobial potential, making them promising candidates for therapeutic development.

### pLANs-producing *S. salivarius* SALI-10 exhibits an extended antimicrobial spectrum against pathogens

**Fig. 4:**
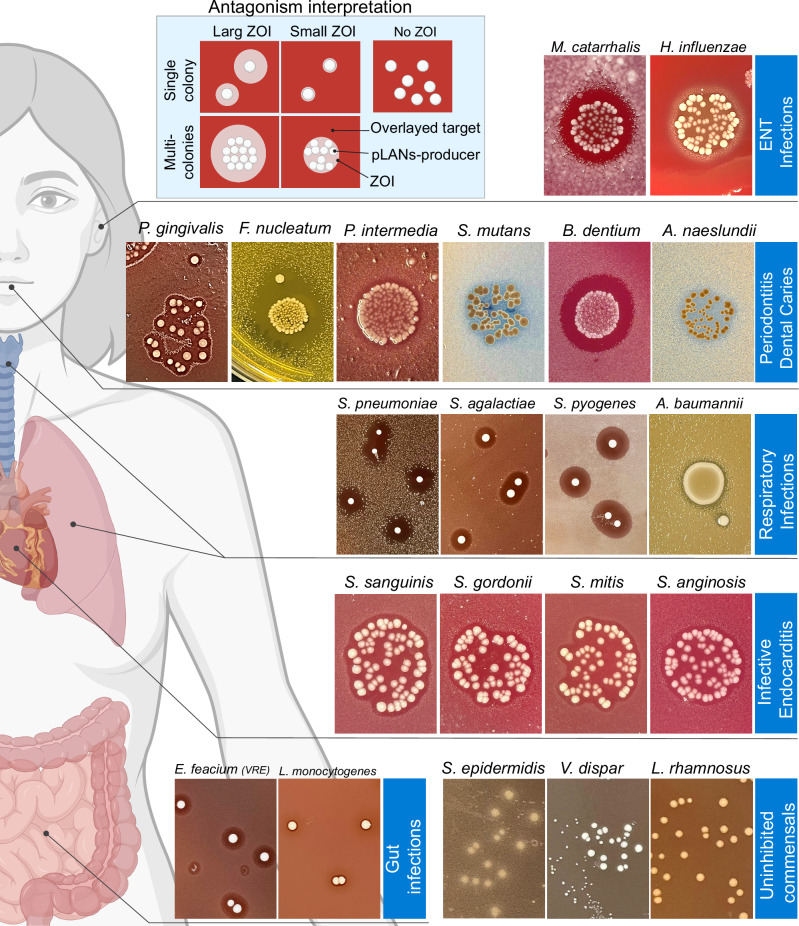
Antagonistic effects of pLANs-producing *S. salivarius* SALI-10 against human-derived bacterial species. An agar diffusion assay was performed to assess the antimicrobial activity of *S. salivarius* SALI-10 against bacterial pathogens implicated in various infections, including ear, nose, and throat infections (ENT), periodontitis, dental caries, upper respiratory tract infections (URIs), pneumonia, infective endocarditis (IE), and gut infections. In this assay, the pLANs-producing *S. salivarius* strain was spotted onto agar media at varying concentrations. After incubation, the colonies were overlaid with molten agar inoculated with the target pathogens. Commensal bacteria, which were unaffected by the strain, were also included as controls. Part of this figure was created in BioRender. Barbour, A. (2026) https://BioRender.com/3e0yfsh.

We selected *S. salivarius* SALI-10 as a model strain for producing pLANs, due to its safety profile^26^, to assess its antimicrobial properties using a deferred overlay antagonism assay against several human microbiome pathogens. SALI-10 exhibited significant growth inhibition against MDR respiratory pathogens, including *S. pneumoniae*, *S. agalactiae*, and *S. pyogenes*, with individual colonies producing substantial zones of inhibition. Notably, SALI-10 also exhibited enhanced inhibitory effects against *Acinetobacter baumannii* in higher-density bacterial cultures, suggesting a concentration-dependent activity (Fig. 4). Additionally, SALI-10 inhibited MDR gut pathogens, including *Listeria monocytogenes* and a VRE strain. In otitis media, SALI-10 inhibited Gram-negative pathogens such as *Moraxella catarrhalis* and *Haemophilus influenzae*, although inhibition was more effective in the presence of multiple producer colonies. SALI-10 also displayed potent antimicrobial effects against Gram-negative periodontal pathogens, including *P. gingivalis*, *Fusobacterium nucleatum*, and *Prevotella intermedia*. Its antimicrobial activity extended to cariogenic species, including *Streptococcus mutans*, *Bifidobacterium dentium*, and *Actinomyces naeslundii*. In contrast, several non-pathogenic commensals from the *Bacillota* phylum, including *Lacticaseibacillus rhamnosus*, *Veillonella dispar*, and *Staphylococcus epidermidis*, were not inhibited by SALI-10 (Fig. 4). These findings align with previous studies on the targeted antimicrobial action of pLANs, particularly against MDR pathogens like *E. faecium* and *S. pneumoniae*^18,19^. The spectrum of salivaricin 10 produced by SALI-10 extends to include viridans streptococci, including *S. sanguinis*, *S. mitis*, *Streptococcus gordonii*, and *Streptococcus anginosus*, which are oral commensals/accessory periopathogens involved in infective endocarditis^27,28^. These findings suggest that SALI-10 has a highly active biosynthetic potential, making it a promising candidate for therapeutic applications against MDR and oral/respiratory pathogens.

### pLANs-producing *S. salivarius* SALI-10 has a strong adhesion phenotype and harbors an arsenal of cell wall-anchored adhesins

**Fig. 5:**
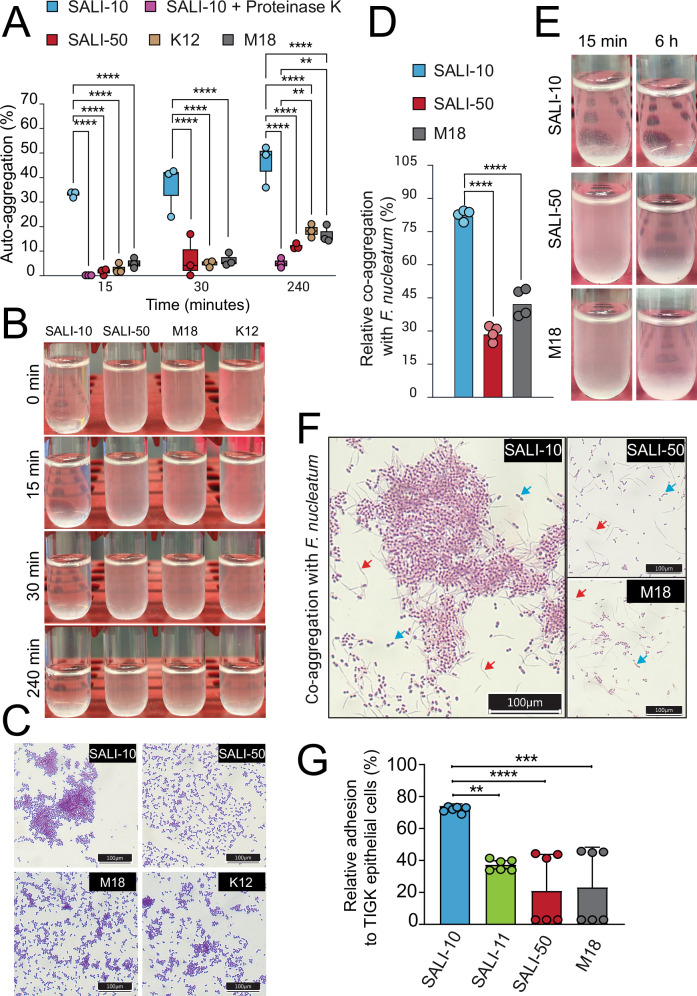
Auto- and co-aggregation phenotype of *S. salivarius* SALI-10. **A** Quantification of auto-aggregation in *S. salivarius* strains. Bacterial cells were washed and suspended in aggregation buffer, and A_600_ was measured at defined timepoints (Methods). Two-way ANOVA with Bonferroni post hoc test was used to compare SALI-10 to other *S. salivarius* strains. **B** Representative photographs of the auto-aggregation assay showing different *S. salivarius* strains sedimenting by time in aggregation buffer. **C** Representative light microscopy images showing distinct auto-aggregation phenotypes among tested *S. salivarius* strains. **D** Represetative graphs of the co-aggregation assay between different *S. salivarius* strains and *F. nucleatum* after 6 h. **E** Representative photographs of the co-aggregation assay showing sedimentation by time in aggregation buffer. **F** Representative light microscopy images showing co-aggregation between *S. salivarius* strains and *F. nucleatum*. Blue and red arrows indicate *S. salivarius* and *F. nucleatum* morphologies, respectively. **G** Adhesion of *S. salivarius* SALI-10 to TIGK epithelial cells, demonstrating its adhesive capacity in comparison to other *S. salivarius* strains.

To produce antimicrobial activity via pLANs in human microbiomes, the therapeutic strain must first adhere to oral surfaces. Since auto-aggregation is a favorable characteristic, deemed an adhesion process influenced by cell surface proteins in *S. salivarius*^29^, we first examined the aggregation phenotype of *S. salivarius* SALI-10 in comparison to other lantibiotic-producing strains. SALI-10 displayed the highest auto-aggregation capacity among those tested. Within 15, 30, and 240 min, approximately 35, 40, and 50% of SALI-10 cells settled from suspension, respectively, about twice the level observed in strains M18 and K12 (~19%) (Fig. 5A–C). These values approach the upper limit previously reported for *S. salivarius* isolates (40–50%)^29^. After 24 h, SALI-10 cultures developed visible sedimentation with a clear supernatant, indicating strong self-aggregation. A lantibiotic-negative strain, namely SALI-50, showed minimal aggregation under identical conditions. Treatment of SALI-10 cells with Proteinase K abolished auto-aggregation, suggesting a key role for surface proteins in mediating this phenotype (Fig. 5A).

We next asked whether SALI-10 could co-aggregate with *F. nucleatum*, an oral species known to interact with certain aggregating *S. salivarius* strains like JIM8777^29^ but not with K12 or M18^30^. SALI-10 strongly co-aggregated with *F. nucleatum*, reaching ~80% aggregation within 6 h and showing clear interaction even at 15 min (Fig. 5D–F). In contrast, strains SALI-50 and M18 displayed only moderate co-aggregation (30–35%) after 6 h, with no detectable interaction at early timepoints.

Finally, SALI-10 exhibited strong adhesion to human gingival keratinocyte (TIGK) monolayers, with over 75% of cells attached to the epithelial cells. Strain M18 showed weak and inconsistent adhesion (~20%), while SALI-11 displayed moderate and reproducible binding (~40%) (Fig. 5G). Together, these results indicate that SALI-10’s enhanced auto- and co-aggregation phenotypes are coupled with superior epithelial adhesion.

**Fig. 6:**
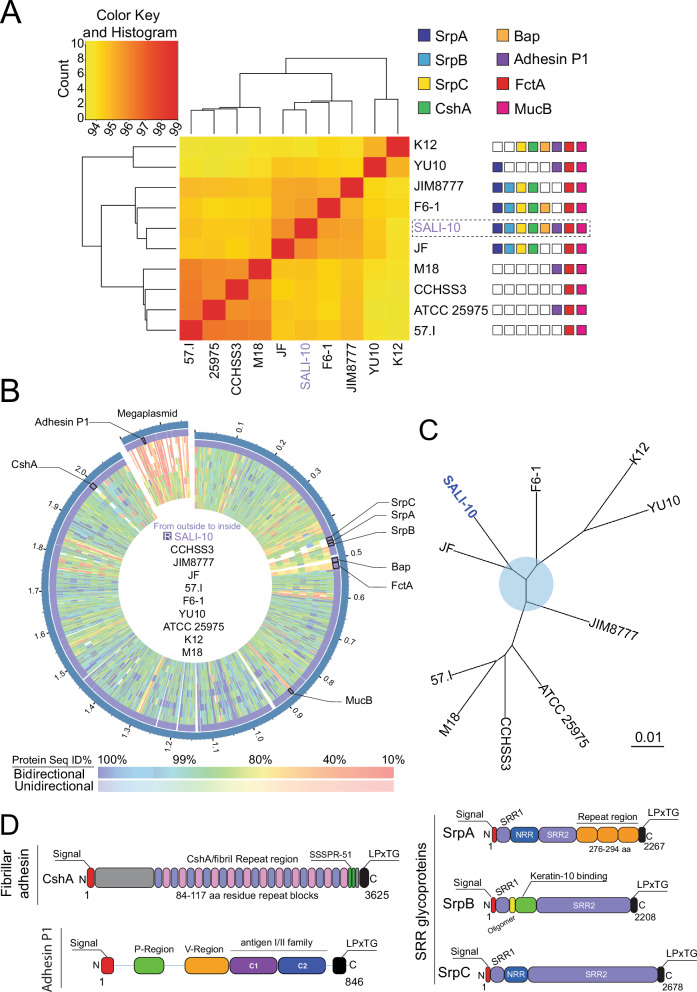
Adhesive surface proteins represent significant genomic variations in pLANs-producing *S. salivarius*. **A** Genomic relationships among *S. salivarius* strains. Hierarchical clustering analysis was conducted based on pairwise genome sequence identity. Conservation of adhesins across the different S. salivarius genomes is indicated. SrpA (dark blue), SrpB (light blue), SrpC (yellow), CshA (green), Bap (Orange), Adhesin P1 (purple), FctA (red), and MucB (magenta). An empty box means that no ortholog was found in the strain genome. **B** Protein sequence-based genome comparison of *S. salivarius* strains via bidirectional BLASTP. S. salivarius SALI-10 served as the reference genome. **C** Phylogenetic reconstruction of the *S. salivarius* genomes utilizing the codon tree method. A dataset comprising 1000 single-copy genes per genome was employed, and comparisons were performed using the Randomized Accelerated Maximum Likelihood approach. **D** Domain architecture of key surface adhesins in *S. salivarius* SALI-10. CshA includes an N-terminal YSIRK signal peptide (red), a non-repetitive region (orange), 24 repeats (pink/purple), an SSSPR-51 domain (green), and a C-terminal LPXTG motif (black). Adhesin_P1 includes Adhesin_P1_N (pfam18652, P-region), BspA variable domain (pfam18220, V-region), and antigen I/II family C terminus (NF033804, C1 and C2). SRR glycoproteins (SrpABC) share a common architecture: signal peptide, two SRR domains, a predicted binding domain (e.g., keratin-10-binding in SrpB), and an LPxTG sorting motif.

Previous studies have comprehensively characterized *S. salivarius*-specific cell wall-anchored adhesins that are essential for adhesion and colonization within the human oral cavity. We conducted a comparative genomic analysis of 10 well-characterized *S. salivarius* strains to elucidate strain-specific differences further. This analysis revealed significant strain-level genomic variation. Pairwise average nucleotide identity (ANI) analysis showed that SALI-10 exhibits the highest similarity with strains JF (96.53%), F6-1 (95.92%), and JIM8777 (95.71%), indicating a close genetic relationship (Fig. 6A). The mean nucleotide divergence within strain clusters ranged from 3% to 6%. In comparison, divergence between clusters reached up to 6%, supporting the presence of genomic subgroups within *S. salivarius*. Furthermore, our analysis confirmed the genomic proximity between strains 57.I, M18, CCHSS3, and ATCC 25975, aligning with previous findings^31^. SALI-10 exhibited the lowest similarity with K12 (94.14%), ATCC 29575 (94.99%), and YU10 (94.98%), indicating that these strains are the most distant from SALI-10 (Fig. 6A).

To identify key proteins contributing to strain-specific functions in SALI-10, we first filtered for the largest SALI-10 proteins absent in at least two other strains. Further functional characterization revealed enrichment for adhesion-related proteins, including the fibrillar adhesins CshA/CshB, biofilm-associated proteins (BAPs), and serine-rich repeat (SRR) glycoproteins (Fig. 6A), suggesting a potential role in host colonization and biofilm formation. Notably, five of the ten strains analyzed, SALI-10, K12, M18, and YU10, as well as ATCC 25975, contained a megaplasmid ranging in size from approximately 160–220 kbp. Genes associated with these megaplasmids included one Adhesin P1-like (adhesion), lantibiotic biosynthesis systems (defense), and adenine-specific DNA methylase (recombination and repair).

Next, we performed a comparative genomic analysis based on protein sequences using bidirectional BLASTP and SALI-10 as the reference genome. We identified sequence gaps or low sequence similarity in several large proteins unique to SALI-10 (Fig. 6B). Phylogenetic reconstruction of the *S. salivarius* genomes showed that SALI-10 is closely related to other *S. salivarius* strains with enrichmed adhesive proteins genes, including JIM8777 and F6-1 (Fig. 6C).

Together with these phenotypic traits, genomic analysis revealed that SALI-10 encodes multiple adhesion systems that likely underpin its strong colonization ability. Such features are absent or divergent in related probiotic strains such as K12 and M18, suggesting strain-specific adaptations for epithelial binding and biofilm stability. Important SALI-10 adhesion proteins are listed in (Fig. 6D), and a complete bioinformatic analysis of these adhesion proteins, including comparative genomics, structural modeling, and phylogenetic relationships, is provided in Supplementary Materials and (Fig. S4).

### Sorbitol metabolism overcomes catabolite repression and enhances pLANs expression in *S. salivarius*

Metabolic profiling revealed that pLANs-producing *S. salivarius* strains are uniquely able to ferment the sugar alcohol sorbitol, distinguishing them from other *S. salivarius* strains (Table S2).

Previous studies indicated that the expression of lantibiotic salivaricins, including pLANs, is influenced by catabolite repression, with mature peptide secretion occurring on solid media but not in liquid cultures^18,32^. As a result, scalability for translational medicine may be challenging, requiring innovative microbial technologies to overcome these limitations. Given the conserved sorbitol metabolism in salivaricin 10 producers, we hypothesized that sorbitol fermentation might activate the *srn* operon transcription and enhance salivaricin 10 production in liquid cultures by overcoming catabolite repression.

**Fig. 7:**
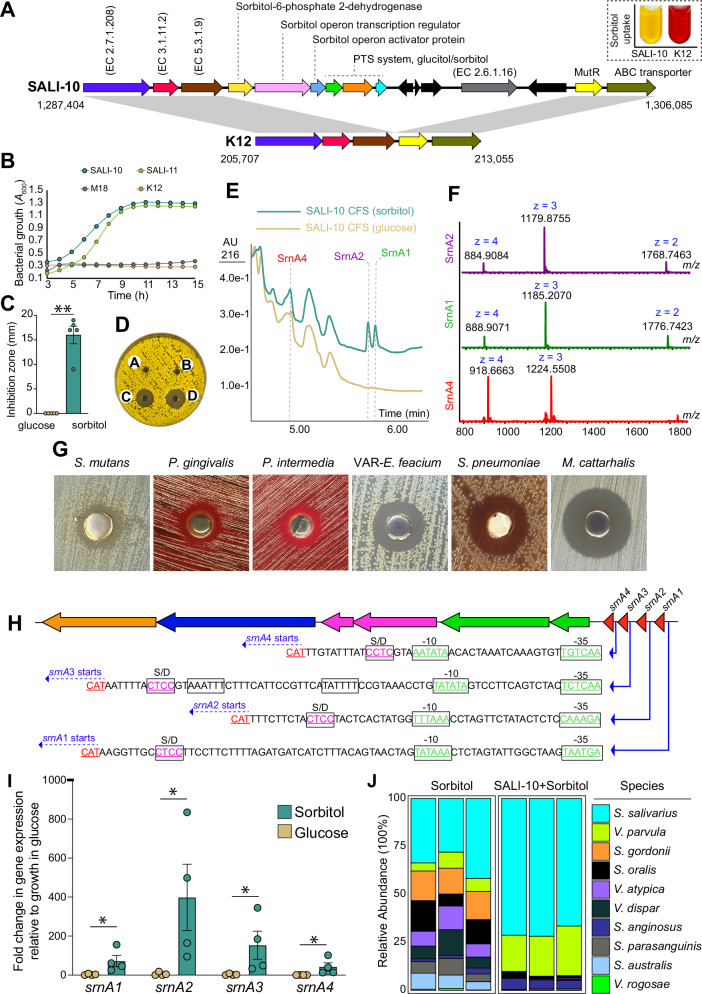
Sorbitol metabolism is a strain-specific trait that enhances pLANs expression in *S. salivarius* SALI-10. **A** Comparative genomic analysis of the sor operon encoding sorbitol uptake in the SALI-10 genome compared to K12, black arrows (genes) indicate mobile elements. The dashed box shows sorbitol utilization by SALI-10 (yellow, positive) or K12 (red, negative). **B** Growth curve analysis of *S. salivarius* strains using sorbitol as the sole carbon source. **C** Antibacterial activity of CFS of strain SALI-10 grown either in the presence of glucose or sorbitol. Well diffusion assay was used with M. luteus as the target strain, and inhibition zones were measured in millimeters. **D** Representative plate from the well diffusion assay. **E** HPLC analysis of CFS obtained from *S. salivarius* SALI-10 grown for 18 h in either glucose or sorbitol media. The three peptides composing salivaricin 10 (peaks SrnA1, SrnA2, and SrnA4) are indicated. **F** MS analysis showing characteristics of SrnA1, SrnA2, and SrnA4, identified directly from CFS of S. salivarius SALI-10 grown with sorbitol as the carbon source. **G** Representative well diffusion assay of chloroform-extracted, C18 SPE purified salivaricin 10 from the CFS of *S. salivarius* SALI-10 grown with sorbitol. Final peptide fraction concentration of 100 µg was used in each well. Zone of inhibition surrounding the wells indicated the antimicrobial activity of pLANs. **H** srn BGC encoding for pLANs expression. We have identified putative promoter regions for each structural gene. **I** Expression of *srnA*_1-4_ was assessed by qRT-PCR in *S.* *salivarius* SALI-10 cells grown in either glucose or sorbitol. **J** Human saliva-derived multispecies biofilms were developed ex vivo either with sorbitol alone or with SALI-10. Shotgun metagenomics data show that sorbitol and SALI-10 treatments caused significant dominance of SALI-10 within the multispecies biofilm.

Comparative genomic analysis of sorbitol-positive SALI-10 and sorbitol-negative strain K12 identified a specialized sorbitol uptake operon (*sor*) in SALI-10, which comprises genes encoding sorbitol-6-phosphate 2-dehydrogenase, sorbitol operon transcription regulator, sorbitol operon activator protein, and three components of the glucitol/sorbitol PTS system (Fig. 7A). Growth analysis of SALI-10 in a minimal medium containing sorbitol alone or in combination with glucose or sucrose showed no significant differences in growth rates (Fig. S5). However, the cell-free supernatant (CFS) from cultures grown with sorbitol alone exhibited potent inhibitory activity, unlike that from cultures grown with combined sugars (Fig. S6). Growth curve analysis also indicated that non-pLAN producers (K12 and M18) were unable to grow in media containing sorbitol as the sole carbon source, unlike pLAN producers (SALI-10 and SALI-11) (Fig. 7B).

Additionally, after 24 h of growth in sorbitol-supplemented medium, CFS obtained from SALI-10 cultures demonstrated potent antimicrobial activity, unlike the same cultures grown in glucose-supplemented medium (Fig. 7C, D). To determine if the observed inhibitory activity correlated with increased pLANs production, we conducted UPLC-QTOF-MS analysis on the CFS and confirmed the extracellular expression of pLANs (SrnA1, SrnA2, and SrnA4) in CFS obtained from the sorbitol cultures. However, only SrnA4 was expressed in the glucose cultures (Fig. 7E, F). The CFS derived from sorbitol fermentation was subjected to chloroform extraction and solid-phase chromatography (refer to Table S3 for specific activity, yield, and purification fold), and the resulting extract was subsequently evaluated for antimicrobial activity using a well-diffusion assay. The purified extract exhibited potent inhibitory activities against clinically significant human pathogens, including *S. mutans*, *S. pneumoniae*, VRE, *M. catarrhalis*, *P. gingivalis*, and *P. intermedia* (Fig. 7G).

Bioinformatics analysis of salivaricin 10 BGC confirmed that each pLANs structural gene (*srnA*_1-4_) has its own independent upstream putative promoter region (Fig. 7H). Gene expression analysis confirmed that sorbitol fermentation resulted in significant overexpression of pLANs structural genes compared to glucose (Fig. 7I).

Multispecies biofilms derived from human saliva were used to investigate the effects of sorbitol on SALI-10 colonization in a static biofilm model. While sorbitol alone encouraged the growth and biofilm formation of *Streptococcus* and *Veillonella* species, the combination of SALI-10 with sorbitol significantly favored the dominance of *S. salivarius* in the biofilm, comprising over 70% of the total bacterial species (Fig. 7J).

### pLANs-producing *S. salivarius* inhibits the pathogenic dental multispecies biofilms and alters the functional metagenome

We previously established a robust, saliva-derived multispecies biofilm model to evaluate the antimicrobial and antibiofilm activities of pLANs in an ex vivo environment^18^. In the current manuscript, we modified this model by inducing dysbiosis through the introduction of the periodontal pathogen *P. gingivalis*^33^, allowing biofilm development under anaerobic conditions for 72 h in the absence (control) or presence (treatment) of only a single dose of the pLANs-producing strain *S. salivarius* SALI-10.

**Fig. 8:**
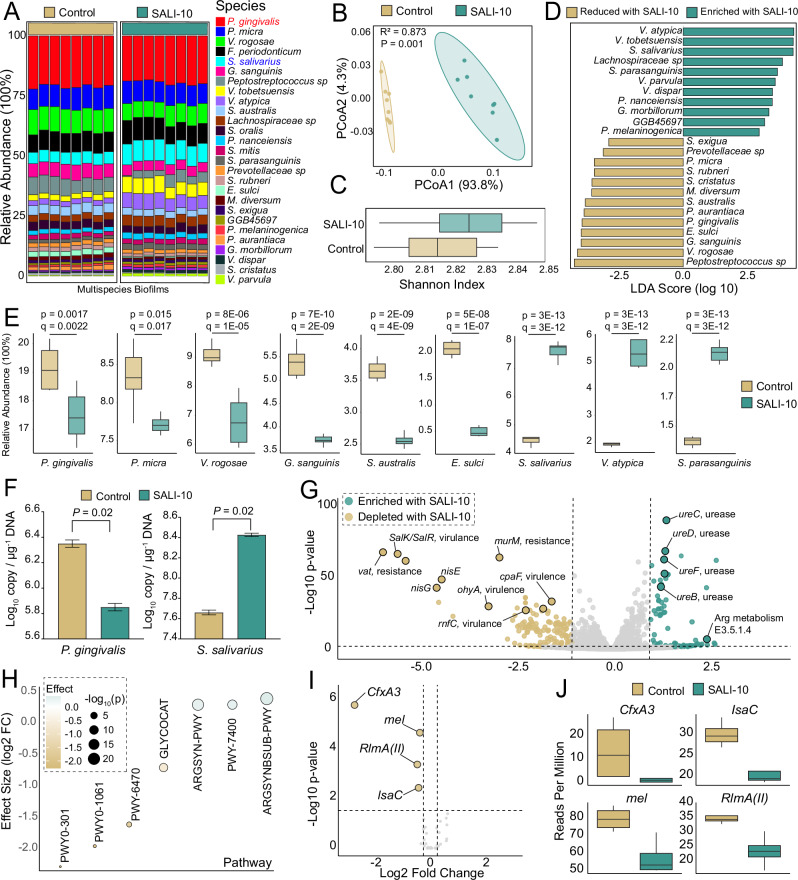
pLANS-producing *S. salivarius* modulates the dental biofilm and prevents periodontal dysbiosis. Multispecies dental biofilms derived from human saliva were inoculated with *P. gingivalis* to induce dysbiosis (control), while *S. salivarius* SALI-10 was added to assess biotherapeutic effects. **A** Taxonomic composition of pathogenic multispecies biofilms with or without SALI-10 treatment. **B** Principal coordinate analysis (PCoA) plot depicting differences in bacterial community composition between control and SALI-10-treated groups. **C** Shannon diversity index of the bacterial communities in control versus SALI-10 groups. **D** Linear discriminant analysis (LDA) identifying bacterial species significantly reduced or enriched by SALI-10 treatment. **E** Box plots showing the relative abundance of bacterial species significantly affected by SALI-10, with statistical significance determined by MaAsLin2 analysis. **F** Quantitative PCR (qPCR) analysis validating the reduction of *P. gingivalis* and enrichment of *S. salivarius* in biofilms following SALI-10 treatment. **G** Volcano plot from functional metagenomics analysis demonstrating altered biofilm functional profiles in response to SALI-10. **H** Significantly affected functional pathways following SALI-10 treatment. **I**, **J** Antibiotic resistance gene analysis showing significant reductions in resistance gene abundance in SALI-10-treated biofilms.

Shotgun metagenomics analysis identified 15 bacterial species with relative abundances >1% in the pathogenic biofilm. *P. gingivalis* dominated (~25%), followed by *Veillonella rogosa* (~13%), *Parvimonas micra* (9%), *Gemella sanguinis* (~7%), *S. salivarius* (~6%), and *Streptococcus australis* (~5%) (Fig. 8A). SALI-10 treatment induced a significant shift in the microbial composition (Bray-Curtis dissimilarity, *P* < 0.05) (Fig. 8B), while alpha diversity (Shannon index) remained unchanged between groups (Fig. 8C). LDA effect size (LEfSe) demonstrated that SALI-10 treatment significantly reduced the abundance of periodontal genera, including *Porphyromonas*, *Parvimonas*, *Prevotella*, and *Peptostreptococcus*. Simultaneously, it facilitated the growth of important oral health-related groups such as *Streptococcus* and *Veillonella* (Fig. S7). These findings confirmed the specific antimicrobial properties of pure pLANs in similar multispecies biofilm settings^18^.

At the species level, a single dose of SALI-10 significantly decreased *P. gingivalis* and five other species (*P. micra*, *V. rogosae, G. sanguinis*, *Eubacterium sulci*, and *S. australis*), while promoting beneficial commensals such as *S. salivarius*, *S. parasanguinis*, and four *Veillonella* species: *V. atypica*, *V. tobetsuensis*, *V. parvula*, and *V. dispar*. (Fig. 8D, E). These findings align with our previous work, which demonstrated that pLANs selectively inhibit disease-associated oral pathogens and promote commensal colonization^18^. Quantitative PCR confirmed a ~1-log reduction in *P. gingivalis* copy number following SALI-10 treatment, with a corresponding 1-log increase in *S. salivarius* (Fig. 8F).

Functional metagenomics analysis further revealed significant shifts in metabolic functions (Fig. 8G). Notably, SALI-10 treatment reduced genes associated with bacterial virulence, including *salK*/*salR* (Signal Transduction), *Streptococcus* virulence [33], *rnfC* (coaggregation, biofilm formation), *Fusobacterium nucleatum* virulence^34^, *cpaF* (tight adherence, Tad pili), *A. actinomycetemcomitans* virulence^35,36^, and *ohyA* (oleate hydratase) *S. pyogenes* virulence^37^. SALI-10 treatment also depleted genes encoding enzymes associated with antimicrobial resistance, including vaT (Virginiamycin A acetyltransferase in *Staphylococcus aureus* and *E. faecium*) and *murM* (*β*-lactam resistance in *S. pneumoniae*)^38^ (Fig. 8G). Conversely, genes associated with urease and arginine deiminase (ADI) pathways were significantly enriched with SALI-10 presence (Fig. 8G). Oral streptococci, including *S. salivarius*, utilize these systems to convert arginine into ammonia, thereby raising the local pH of biofilms and restricting acid-induced enamel demineralization by mutans streptococci, the causative agents of tooth decay^39^.

Following SALI-10 treatment, metagenomics pathway analysis of the multispecies biofilm revealed significant changes in metabolic pathways. A total of 23 pathways were upregulated, while 12 were downregulated (FDR < 0.05). Among the upregulated pathways, there was notable enrichment in L-arginine biosynthesis (ARGSYNBSUB-PWY, ARGSYN-PWY, PWY-7400) (Fig. 8H). These alterations suggest increased alkali production and decreased acidogenic potential. On the other hand, pathways associated with virulence and dysbiosis were downregulated. Glycogen degradation I (GLYCOCAT-PWY), which contributes to prolonged acid production in cariogenic species, and L-alanine biosynthesis (PWY0-1061), essential for peptidoglycan synthesis, were both inhibited. Notably, the pathway related to *β*-lactam resistance (PWY-6470) was also decreased, suggesting a potential reduction in traits associated with antimicrobial resistance. Furthermore, a drop in vitamin C degradation (PWY0-301) and peptidoglycan turnover indicates decreased pro-inflammatory and tissue-damaging microbial activities (Fig. 8H).

Resistome analysis via the Comprehensive Antibiotic Resistance Database (CARD)^40^ demonstrated that SALI-10 treatment eliminated the *β*-lactam resistance gene *cfxA* from the biofilm (Fig. 8I, J). The *cfxA* gene family, previously detected in periodontal pockets, is predominantly carried by *Prevotella*, *Porphyromonas*, and *Bacteroides*^41^. Additionally, the abundance of the macrolide resistance gene *mel*, commonly found in *Streptococcus* and *Gemella* spp. and transferable to the pathogen *S. pneumoniae*^42^, was significantly reduced following SALI-10 intervention (Fig. 8I, J).

These findings highlight the potential of pLANs-producing *S. salivarius* SALI-10 to modify pathogenic dental biofilms by selectively inhibiting key pathogens and diminishing virulence and antibiotic-resistance factors. This reinforces its promise as a therapeutic approach to prevent oral dysbiosis.

### *S. salivarius* SALI-10 integrates into the dental multispecies biofilms and changes their biosynthetic profile

**Fig. 9:**
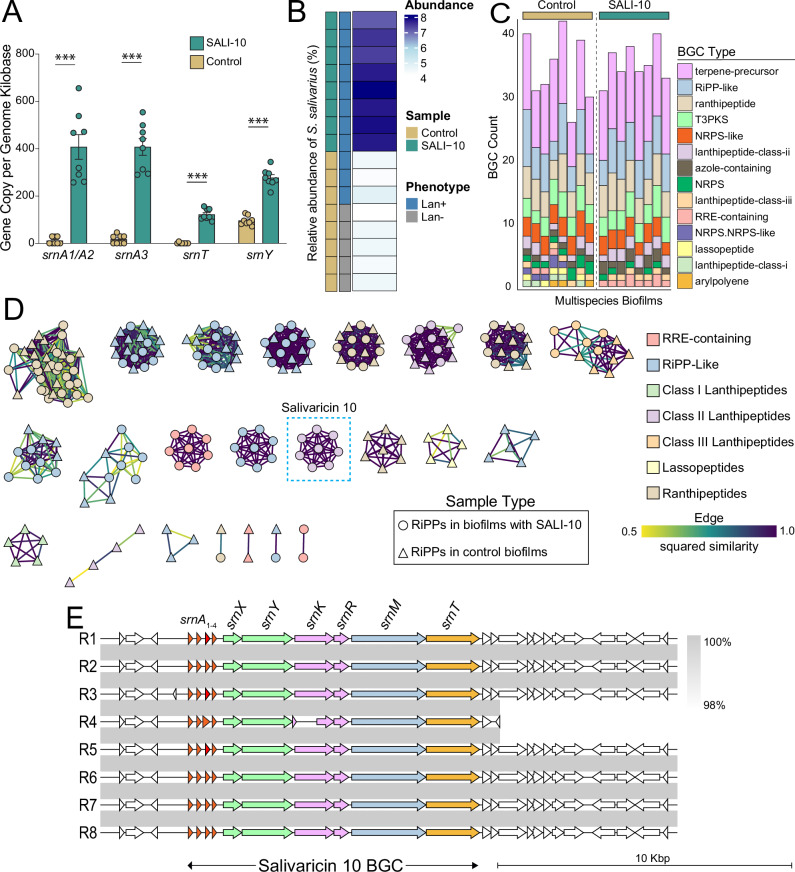
pLANs-producing *S. salivarius* integrates into the multispecies biofilm and alters its biosynthetic potential. **A** Salivaricin 10 BGC genes (srn) were extracted from short read sequences (using an 80% similarity cut-off) and compared across multispecies biofilms. SRN genes had significantly higher gene copy number per genome kilobase in SALI-10-supplemented biofilms than in controls. **B** Metagenomic data were used to analyze two phenotypes of *S. salivarius* in the biofilms based on lantibiotic encoding genes (Lan^+^ and Lan^-^). Endogenous control biofilms predominantly contained (Lan^-^) *S. salivarius* phenotypes, whereas SALI-10-supplemented biofilms showed a dominance of (Lan^+^) phenotypes with a significant increase in species relative abundance. **C** The biosynthetic potential of multispecies biofilms was assessed using assembled metagenomes, and BGCs were compared. **D** Similarity networks based on the assembled metagenomes confirmed that the salivaricin 10 BGC was present only in SALI-10-supplemented biofilms, indicating successful integration of the strain and modification of the biosynthetic potential of the biofilm. **E** The salivaricin 10 BGCs were retrieved from the similarity network and compared across replicates, showing successful organization of the locus in all SALI-10-supplemented biofilm replicates (*n* = 8).

To assess whether *S. salivarius* SALI-10 successfully engrafts into the ex vivo dental multispecies biofilm and expresses its signature lantibiotic pathway, we performed strain-specific analysis of the pLANs BGC. Using shotgun metagenomics sequencing, we extracted reads corresponding to the pLANs biosynthetic genes (*srnA1-4, srnX, srnY, srnR, srnK, srnM*, and *srnT*) via nucleotide BLAST (with a sequence similarity threshold of ≥80%). Among these, *srnA*_1-3_ (lantibiotic precursors), *srnT* (transport), and *srnY* (immunity) were detected and showed significantly higher normalized gene abundance in SALI-10-treated biofilms compared to controls (Fig. 9A). To characterize the multispecies biofilm BGCs more broadly, we generated short-read assemblies and screened for natural product pathways using antiSMASH. We identified lanthipeptide-positive regions based on annotation and domain structure. In control biofilms, two phenotypes of *S. salivarius* (lanthipeptide-positive and lanthipeptide-negative) were present at approximately 2% relative abundance each. In contrast, SALI-10-treated biofilms displayed a complete shift, with 100% of *S. salivarius* reads corresponding to the lanthipeptide-positive genotype (Fig. 9B), indicating the engraftment of the introduced SALI-10 and the displacement of the indigenous strain variants.

Further analysis using the BiG-SCAPE pipeline confirmed the diversity of biosynthetic potential within the biofilm community. More than 20 unique BGCs related to RiPPs and non-ribosomal peptide synthetases (NRPS) were identified in each sample, highlighting a chemically rich microbiome (Fig. 9C). Among these, classes I, II, and III of lanthipeptides, as well as thiopeptides and ranthipeptides, were detected.

The entire salivaricin 10 BGC was detected only in samples treated with SALI-10. In contrast, it was absent in control biofilms (Fig. 9D). Structural validation confirmed the domain architecture and the integrity of the BGC, aligning with a functional pathway (Fig. 9E). Collectively, these results indicate that SALI-10 not only integrates into the biofilm community but also enhances the biosynthetic potential of the microbiome.

### Transplantation of SALI-10 reduces periodontal pathogens and oPMN counts in the human oral cavity

**Fig. 10:**
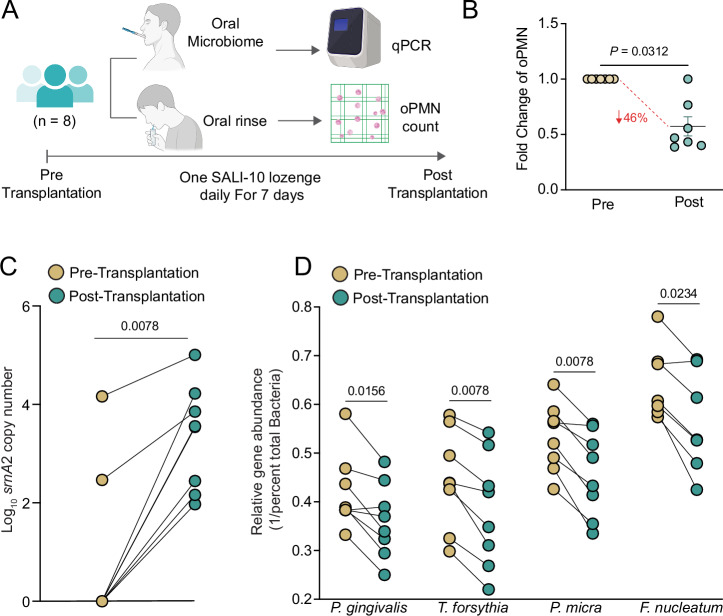
Transplantation of pLANs-producing *S. salivarius* reduces oPMN counts and periodontal pathogens in the human oral cavity. **A** Human subjects (*n* = 8) consumed one lozenge per day for 7 days containing 10^10^ CFUs of *S. salivarius* SALI-10. Oral microbiome and oral rinse samples were collected at baseline (pre-transplantation) and after 7 days of lozenge use (post-transplantation). **B** Quantification of the oPMN counts measured in oral rinses collected at Pre- and Post-transplantation. Data expressed as fold change. **C** *srnA*2 copy number in the oral cavity was quantified by qPCR analysis. **D** qPCR-derived relative abundance of periopathogens. Data are presented as reciprocals of the mean normalized cycle threshold (CT) values (CT_periodontal pathogen_/CT_total bacteria_). Statistical analysis was based on the non-parametric Wilcoxon matched-pairs signed-rank test. *0.01 < *P* < 0.05; **0.001 < *P* < 0.01. Part of this figure was created in BioRender. Barbour, A. (2026) https://BioRender.com/tj6f125.

Since SALI-10 successfully integrated into ex vivo-grown multispecies biofilms and demonstrated inhibitory effects against periodontal pathogens, we sought to assess its efficacy in performing similarly in humans’ feasibility pilot trial. We have created easy-to-dissolve oral lozenges, each containing ~1 × 10^10 ^c.f.u of *S. salivarius* SALI-10, and eight healthy human subjects consumed a lozenge per day for 1 week. Oral microbiome samples and oral rinse samples were collected at baseline (pre-transplantation) and after 7 days of lozenge use (post-transplantation) (Fig. 10A). To evaluate short-term changes in host inflammatory burden, oPMN counts in oral rinse samples were quantified^43–47^ before and after SALI-10 supplementation. Following the 7-day intervention, 46% reduction in oPMN counts was observed (Fig. 10B).

Colonization dynamics following SALI-10 administration were assessed using qPCR targeting the *srnA*2 gene, a marker of the *srn* BGC. Prior to intervention, *srnA*2 was undetectable in 6 of 8 subjects; however, following lozenge consumption, all eight subjects exhibited detectable *srnA*2 copy numbers (≥100 copies/mL) (Fig. 10C). The remaining two subjects showed further increases relative to baseline. Enrichment of a*n SRN A*2-harboring population suggests consistent engraftment of the administered strain.

We next evaluated pathogen suppression by quantifying changes in the abundance of disease-associated taxa. Relative qPCR analysis of oral samples revealed significant reductions in key periodontal pathogens following the intervention. Members of the red complex, *P. gingivalis* and *T. forsythia*, were significantly reduced post-treatment (*P* < 0.05 and *P* < 0.01, respectively). At the same time, *T. denticola* remained below detection in all samples (Fig. 10D). Additionally, members of the orange complex, *P. micra* and *F. nucleatum*, were also significantly decreased (*P* < 0.01 and *P* < 0.05, respectively).

Together, these observations are consistent with the ex vivo findings and indicate that SALI-10 can engraft within the human oral microbiome over a short-term intervention, coinciding with suppression of disease-associated taxa and a reduction in oPMN counts. While limited by sample size and study duration, these results support the feasibility of using metabolically and functionally adapted commensal streptococci to modulate oral microbial communities in vivo.

## Discussion

Over the past decade, natural product-producing commensals have attracted increasing attention as an alternative to antibiotics for combating disease-causing bacteria, particularly in this era of rising antibiotic resistance^48,49^. Due to its ability to exert targeted antimicrobial effects against MDR pathogens, *S. salivarius* has emerged as a promising candidate for probiotic and biotherapeutic development^50^. As a predominant inhabitant of the tongue, oral buccal surfaces, and saliva, it plays a crucial role in shaping the oral microbiome, contributing to host health through microbiome stabilization and pathogen exclusion^16^.

In this study, we developed a screening approach to isolate beneficial *S. salivarius* strains with robust biosynthetic potential. Under the tested conditions, this strategy consistently identified pLANs as the predominant AMPs produced by this species. The inhibitory activity of the producing strain SALI-10 is attributable to pLANs biosynthesis, as supported by prior genetic and chemical evidence. Since pLANs are encoded on the pSALI-10 megaplasmid, curing the megaplasmid resulted in complete loss of antimicrobial activity. Proteomic analysis of the cured strain revealed an otherwise unchanged metabolomic profile, with the sole exception being the absence of the three pLANs peptides^18^. The selective loss of these peptides precisely coincided with the loss of bioactivity, strongly implicating pLANs as the molecular basis for inhibition.

In addition to its specialized metabolic and antimicrobial capabilities, SALI-10 displays surface features important for persistence in the oral environment. Its strong aggregation behavior supports microcolony formation and stable incorporation into biofilms. Moreover, SALI-10 surface SRR glycoproteins are implicated in glycoprotein-mediated adhesion to epithelial surfaces^51^. A previous study showed that SRR glycoproteins are conserved symbiosis factors and part of the “colonization island,” which is critical for host-specific mucosal colonization in *Bacillota*, including Streptococci and Lactobacilli^52^. These large, glycosylated adhesins mediate high-affinity binding to mucosal glycans and are essential for stable epithelial attachment^52^, and biofilm formation^53^. Notably, the predicted SALI-10 KRT10-binding domain in SrpA protein implicates it in epithelial niche targeting, potentially limiting colonization by respiratory pathogens such as *S. pneumoniae*^54^, offering broader relevance in upper airway colonization resistance^55,56^. Similarly, SALI-10 harbors an adhesin P1 homolog, analogous to *S. mutans* antigen I/II^57^, that may further facilitate competition for oral biofilm adhesion sites.

The low prevalence, strain-specific coexistence of surface adhesion modules in *S. salivarius*, with <5% naturally producing pLANs, indicates functional integration of adhesion and antimicrobial strategies. SALI-10’s enhanced adhesion and pLANs biosynthesis, driven by sugar alcohol metabolism, promote surface persistence and pathogen inhibition. More broadly, strain-level differences in surface adhesins and other cell-envelope proteins can shape mucosal persistence and competitive fitness, providing a rational basis for selecting and engineering higher-performing probiotic strains^58^.

Our findings advance the understanding of how *S. salivarius* dynamically responds to environmental cues to regulate pLANs biosynthesis and modulate host-associated microbial communities. The observed repression of salivaricin 10 BGC expression in glucose and its strong induction in the presence of sorbitol underscores a classical catabolite repression mechanism, likely mediated by CcpA, that prevents costly secondary metabolite production during nutritional abundance^59^. The derepression observed with sorbitol indicates a metabolic sensing pathway that triggers antimicrobial activity under carbon-starved or stressful conditions. This flexible regulation reflects ecological pressures in the oral cavity, with nutrient levels fluctuating in response to diet, salivary flow, and mucosal interfaces. *S. salivarius*’s capacity to connect carbon source availability to lantibiotic production exemplifies a strategic adaptation, conserving energy-intensive biosynthesis for environments where it provides a competitive edge.

In this context, our findings are consistent with emerging ecological models in which successful strain establishment within complex biofilms depends on a combination of metabolic advantage and conditional antimicrobial deployment^60^. Access to sorbitol through a specialized uptake operon may provide an initial growth advantage, enabling population expansion within the resident community. Coupling of sorbitol metabolism to induction of the pLANs BGC may then permit density-dependent antimicrobial activity, providing a plausible framework for the dominance of SALI-10 observed in biofilm models.

Importantly, sorbitol by itself has been shown in multiple clinical studies to have no significant effect on the composition of the human oral microbiome^61–63^, likely due to the homeostatic influence of salivary flow and the inherent resilience of microbial communities in healthy individuals. In contrast, sorbitol functions in the current study as a metabolic cue that selectively enhances pLANs expression in responsive strains such as SALI-10.

In a pilot human study, daily administration of lozenges containing a pLANs-producing strain led to measurable colonization, suppression of red complex and orange complex pathogens, and a reduction in oPMN counts. A limitation of this pilot is that colonization was inferred using a targeted qPCR assay against the *srnA2* gene rather than genome-wide strain tracking. While *srnA2* marks the salivaricin-10 biosynthetic locus, homologous sequences can occur in other oral streptococci, limiting absolute strain-level resolution. Accordingly, the observed signal is interpreted as enrichment of a *srnA2*-harboring population rather than definitive detection of a single clonal strain. Future randomized clinical trials may use shotgun metagenomic sequencing targeting the complete SALI-10 genome as a reference to enable unambiguous strain-level tracking.

We previously demonstrated that gingival immune activity is tightly coupled with neutrophil recruitment into the oral cavity, reflecting the sensitivity of the oral-immune interface to microbial perturbations^64^. Experimental disruption of oral homeostasis, such as cessation of oral hygiene, provokes a rapid increase in oPMN recruitment, consistent with heightened immune surveillance in response to microbial challenge^65^. Intriguingly, we previously found that individuals classified as “slow responders” to experimental gingivitis exhibited significantly higher relative abundance of oral streptococci, suggesting a protective association between commensal streptococcal dominance and restrained immune activation^66^. In this context, colonization with pLANs-producing streptococci may serve as a host-compatible strategy to modulate immune surveillance at the mucosal surface. In the present feasibility study, administration of SALI-10 was associated with a marked reduction in oPMN counts, which we interpret as a decrease in microbiome-driven immune recruitment rather than a direct measure of reduced inflammation. This change is consistent with the observed depletion of disease-associated taxa and suggests a reduced requirement for neutrophil surveillance due to diminished pathogen-associated immune stimulation.

An alternative explanation is that pLANs enhance the efficiency of innate immune surveillance, allowing more effective control of microbial triggers with lower overall cellular recruitment. Together, these findings align with emerging models of microbiome-immune cooperation, in which specific commensals contribute to colonization resistance and immune homeostasis without suppressing protective host responses^67,68^. Building on these foundational findings, randomized, double-blind, placebo-controlled clinical trials are warranted in human disease models, including experimental gingivitis and other oral inflammatory conditions, to evaluate SALI-10’s therapeutic potential through clinical endpoints and immune profiling.

In conclusion, we present evidence for a metabolically adapted subset of *S. salivarius* that produces pLANs to defend its niche and promote oral health. These beneficial microbes utilize adhesive strategies for mucosal colonization and exploit non-preferred sugar metabolism to coordinate the synthesis of pLANs. Daily intake of lozenges containing these beneficial microbes reduced oPMN counts, suppressed pathogenic taxa, and enriched health-associated streptococci, collectively supporting its potential as a precision-targeted probiotic. This study presents a refined model of pLANs regulation and ecological function, introducing a clinically viable therapeutic strategy that bridges the microbial, metabolic, and immunological dimensions of oral health.

## Methods

### The observational clinical study

The study was approved by the Office of Research Ethics at the University of Toronto (protocol number 45841) and was conducted in accordance with the Helsinki Declaration of 1975. All research subjects signed an informed consent form before enrollment and sample collection. This clinical case-control observational study involved two groups: one with PD and the other with healthy individuals. Twenty subjects were recruited, with Group 1 consisting of periodontally healthy subjects (Group H; *n* = 10) and Group 2 comprising subjects with Stage III or Stage IV PD (Group PD; *n* = 10). Detailed patient histories were collected, and two calibrated examiners performed clinical examinations. Subjects’ periodontal diagnoses followed the parameters outlined in the 2017 PD classification by the American Academy of Periodontology. Using this classification, patients were diagnosed with either clinical gingival health on intact periodontium or on reduced periodontium. The criteria for clinical gingival health on intact periodontium include <10% bleeding sites, probing depths ≤3 mm, and no attachment or bone loss. For clinical gingival health on reduced periodontium, criteria include <10% bleeding sites, no probing depths of 4 mm or greater that bleed on probing, and no signs of progressive periodontal breakdown. The inclusion criteria included males and females of any ethnicity aged 18 years or older. Exclusion criteria included pregnancy, recent antibiotic treatment within the last 6 months, current smokers, or subgingival instrumentation within the previous 3 months. Subgingival plaque from healthy and PD patients was collected using an 11/12 Gracey curettes. For subjects with PD, samples were collected from a “diseased” periodontal pocket (defined as one with a depth greater than 4 mm, bleeding on probing, and interproximal attachment/bone loss). For healthy subjects, samples were collected from a “healthy” gingival sulcus (<3 mm, absence of bleeding on probing, no suppuration). The plaque samples were resuspended in 200 µL of PBS, and the DNA was extracted using the DNeasy PowerSoil Pro Kit (Qiagen) according to the manufacturer’s instructions.

### Shotgun metagenomics

Samples containing 90–220 ng of DNA were used as input for library preparation with the Illumina DNA Prep Kit, following the protocol outlined in the Illumina DNA Prep Reference Guide (Document #1000000025416 v10). Six PCR cycles were performed during the Amplify Tagmented DNA step. Adapter-ligated, barcoded libraries were quantified using the Quant-iT dsDNA Assay Kit (Thermo Fisher Scientific) on a Qubit fluorometer and then pooled in equal amounts. The final pooled library was re-quantified with Qubit and diluted according to the Denature and Dilute Libraries for the NextSeq 1000/2000 protocol (Standard SBS Onboard Denature and Dilute). Sequencing was performed on the Illumina NextSeq 2000 using a P1 flow cell (2 × 150 bp) at a final loading concentration of 750 pM. Fastp trimming was used for adapter removal and filtering of low-quality reads using -5 "3" -3 "3" -r "4:15" --dedup parameters with resulting minimum sequence length of 125 bp^69^.

### Microbiome analysis

Bowtie2 and the hg38 database were employed to detect human contamination in the sequences^70^. Unmapped, non-human reads were then analyzed as part of the microbial metagenome. Paired-end reads (R1 and R2) were combined into a single file. Using default settings, the MetaPhlAn 4.1.1 tool was utilized for taxonomic profiling, with the chocophlanvJun23_202403 database and the UniRef90 database for functional annotation^71,72^. The final merged gene families table was organized by KEGG ID. Species-level abundance data from MetaPhlAn were imported into RStudio (R version 4.4.2) and calculated by summing the reads per million (RPM) values and normalizing them to the total RPM per sample, generating relative abundance percentages. Stacked bar plots were created using the ggplot2 package (v3.5.1) to visualize species-level taxonomic composition across all samples^73^. Bray-Curtis dissimilarities were calculated using the vegdist function from the vegan package (v2.6-10)^74^, and principal coordinates analysis (PCoA) was performed and plotted using the cmdscale function and ggplot2. Group-level differences were tested using PERMANOVA by the adonis2 function, and pairwise comparisons were performed with a custom function. Confidence ellipses (95%) were added to visualize group clustering. To identify bacterial taxa significantly altered by SALI-10 treatment, linear discriminant analysis (LDA) was performed. Taxa were filtered based on significance thresholds derived from p-values and false discovery rate (*q*-values). Metagenomics functional profiles were annotated with KEGG Orthology (KO) terms using the KEGGREST package (v1.42.0)^75^. To identify pathway-level functional changes, a volcano plot was constructed using the ggplot2, dplyr (version 1.1.4), ggrepel (version 0.9.5), and plotly (version 4.10.4) packages. Threshold lines were added at |log₂FC | = 1 and −log₁₀(p) = 1.82 (corresponding to *p* = 0.015). log2 fold changes and -log10(*p*-values) were derived from comparisons between SALI-10-treated and control groups. Features were classified as enriched in the SALI-10 group (*P* < 0.05, log₂FC > 1), enriched in the control group (*P* < 0.05, log₂FC < −1), or not significant. To quantify antimicrobial resistance genes (ARGs), the Resistance Gene Identifier (RGI) was used to predict resistomes from short sequencing reads using reference sequences in the CARD. Raw RPM-normalized abundance data for ARGs were imported into R. Differential abundance analysis was performed to calculate log₂FC, raw *p*-values, FDR, and -log₁₀(*p*-values). To quantify the pLANs BGC, Individual salivaricin 10 BGC genes (*srnA*_1-4_, *srnX*, *srnY*, *srnR*, *srnK*, *srnM*, and *srnT*) were queried using NCBI nucleotide BLAST (blastn). Hits with ≥80% sequence identity were retained and imported into R. A combined gene family abundance table was filtered to retain only entries matching the accession numbers of BLAST hits. Accessions were annotated by their corresponding *SRN* gene for downstream analysis. To validate gene identities, protein sequences were aligned to reference Salivaricin 10 BGC genes using UniProt alignment tools. A percentage-identity matrix and a phylogenetic tree were analyzed to determine similarity. Only accessions with ≥80% identity to a known *srn* gene were retained. Non-parametric Kruskal–Wallis tests and Wilcoxon signed-rank tests (with Benjamini–Hochberg correction) were performed to assess differences in copy number across genes.

### Calculation of the relative abundance of Lan-positive and Lan-negative *S. salivarius* phenotypes

To identify different S. salivarius phenotypes in the metagenomes based on the presence or absence of lantibiotic BGCs, assembled metagenomes were first analyzed using the antiSMASH pipeline to identify BGCs, including those encoding lantibiotics. The resulting GenBank files were converted to FASTA format and subjected to NCBI BLASTn for taxonomic classification, using the following filters for top hits: e-value < 1e-3, sequence identity ≥75%, and query coverage ≥ 60%. Contigs identified as *S. salivarius* were then filtered to retain only those containing lantibiotic BGCs. These lantibiotic-positive *S. salivarius* contigs were mapped to species-level taxonomic profiles generated by MetaPhlAn to estimate the relative abundance of *S. salivarius* strains carrying (Lan-positive) or lacking (Lan-negative) lantibiotic gene clusters.

### Age-adjusted microbial differential abundance analysis

Differential abundance analysis of microbial taxa was performed using MaAsLin2. Relative abundance data were modeled using disease status (healthy vs. PD) as the primary fixed effect. To account for potential confounding by age, which differed between clinical groups, a second multivariable model was fit, including age as a continuous covariate. In the covariate-adjusted model, regression coefficients for disease status represent associations independent of age, while coefficients for age represent associations independent of disease status. Only features passing MaAsLin2 default quality control and prevalence thresholds were retained for analysis. Statistical significance was assessed using MaAsLin2-reported p-values, and associations with *p* < 0.05 were considered significant. For visualization, age-associated and disease-associated taxa were summarized in heatmaps in which statistical significance was encoded as -log_10_(p). Significant features were outlined, and the direction of association (positive or negative) was indicated for statistically significant taxa only. This multivariable approach allowed us to distinguish microbial features associated with disease status from those driven by age-related variation.

### Aerobic/anaerobic growth conditions

Bacterial strains that require aerobic growth (Table S4) were grown at 37 °C and 120 rpm. Bacterial strains that require 5% CO_2_ for aerobic growth were grown stationary at 37 °C in a CO_2_ incubator. Bacterial strains requiring anaerobic growth were grown in a stationary state in the BD GasPak™ EZ anaerobic pouch system.

### Antimicrobial assays

An agar antagonism assay was developed to investigate the secretion and antagonistic activity of active salivaricin 10 peptides from the producer strain against targeted pathogens. *S. salivarius* was grown overnight at 37 °C in TSYE. The culture was then subjected to serial dilutions, and 20 µL of each dilution was spotted onto the appropriate agar plate. The plates were incubated overnight at 37 °C before the grown producer colonies were subsequently overlaid with 5 mL of soft, molten agar at 50 °C, supplemented with 25 µL of a mid-log culture of the target bacteria. Once the agar had solidified, the plates were incubated under the appropriate conditions for the target used (Table S4). Zones of inhibited pathogen growth surrounding the producer colonies were observed. For well-diffusion assays, an adequate volume of target pathogenic cells was spread evenly across the surface of appropriate agar media. A hole with a diameter of 6 mm was aseptically punched using a Pasteur pipette. The supernatant from the producer strain culture was harvested by centrifugation at 17,000 × *g* for 10 min. Then, 100 µL of the supernatant was added to the well and allowed to air-dry. This step was repeated with an additional 100 µL of supernatant. For more purified fractions processed by chloroform extraction and C18 columns, a final concentration of 100 µg of peptide was used in each well. After drying, the plates were incubated under anaerobic conditions to promote the growth of pathogens. Zones of inhibited growth surrounding the well were interpreted as evidence of antimicrobial activity.

### Fluorescent labeling with pHrodo

*S. pneumoniae* ATCC 6301 was grown overnight on tryptic soy agar supplemented with 5% sheep blood at 37 °C aerobically in 5% CO₂. Cells were resuspended in PBS to an optical density at 600 nm (A600) of 0.5. Aliquots of 500 µL were prepared and heat-killed at 70 °C for 10 min. Heat-killed *S. pneumoniae* cells were labeled using the amine-reactive pHrodo™ Green STP ester (Thermo Fisher Scientific, Cat. #P36600) following the manufacturer’s instructions. Briefly, a 500 µg vial of pHrodo™ dye was dissolved in 75 µL dimethyl sulfoxide (DMSO) to yield an ~8.9 mM stock solution. For staining, 25 µL of stock was added to 500 µL of bacterial suspensions (final concentration 0.5 mM) and incubated for 45–60 min at room temperature in the dark. Following incubation, 750 µL Hank’s Balanced Salt Solution (HBSS) was added before centrifugation at 15,000 × *g* for 1 min. The supernatant was removed, and the pellets were resuspended in 1.5 mL 100% methanol, followed by an additional 0.5 mL methanol, vortexing for 30 s, and centrifugation as above. The pellet was washed twice with HBSS (1.0 mL, then 0.5 mL; vortex for 30 s each) to remove unbound dye. Labeled bacteria were resuspended in sterile water to 50 mg/mL and freeze-dried. Lyophilized bacteria were stored at −80 °C until use.

### Phagocytosis assay using whole blood and flow cytometry

Peripheral blood was collected from healthy human donors into sodium citrate tubes. Aliquots of 100 µL whole blood were incubated with AMPs isolated from different *S. salivarius* strains for 10 min at 37 °C. Titrated pHrodo™-labeled *S. pneumoniae* was then added, and samples were incubated for an additional 20 min under the same conditions. Following incubation, erythrocytes were lysed with 1× BD Pharm Lyse buffer (BD Biosciences, Cat# 555899) for 15 min at room temperature. Samples were centrifuged at 400 × *g* for 5 min at 4 °C, and the cell pellet was washed once with cold PBS under the same conditions. Cells were stained with Zombie NIR™ Fixable Viability Dye (BioLegend, Cat# 423106) for 15 min on ice in the dark, followed by washing with FACS buffer (PBS supplemented with 0.09% NaN₃, 0.2% BSA, and 2 mM EDTA). For surface staining, cells were incubated with anti-CD16 Alexa Fluor^®^ 700 (BioLegend) for 30 min on ice in the dark, washed once in FACS buffer, and resuspended for acquisition. Data were acquired on a Sony SA3800 spectral flow cytometer, with a minimum of 20,000 live singlet CD16⁺ neutrophils recorded per sample. Flow cytometry analysis was performed by excluding debris and doublets (FSC/SSC gating), followed by identification of live singlet CD16⁺ neutrophils. The frequency of pHrodo-positive neutrophils was quantified, with thresholds set using titrated pHrodo-labeled *S. pneumoniae* in untreated control samples, and positive gates defined relative to these controls.

### Aggregation assays

*S. salivarius* strains were grown overnight at 37 °C in THYE before the cells were harvested at 3000 × *g* for 10 min and washed twice with phosphate-buffered saline (PBS). The cells were then resuspended in PBS to an *A*_600_ of 0.5. Bacterial suspensions were incubated at room temperature without agitation. At 4 h intervals over 24 h, 200 µL of the upper suspension was carefully removed without disturbing the settled cells, and *A*_600_ was measured using a spectrophotometer. The auto-aggregation percentage was calculated using the following formula:$\,{Auto}-{aggregation}\,( \% )=(1-A600\,{at}\,{time}\,t/A600\,{at}\,{time}\,0)\,\times 100$ where *A*_600_ at time 0 represents the initial optical density, and *A*_600_ at time t represents the optical density at each time point. The results were plotted to show the extent of auto-aggregation over time, with higher percentages indicating greater auto-aggregation. For co-aggregation assays, equal volumes of *S. salivarius* cell suspensions were mixed with *F. nucleatum* ATCC 10953 prepared under identical conditions, or with aggregation buffer alone as a control. Mixed suspensions were incubated at room temperature without agitation for up to 6 h. Co-aggregation was quantified as described previously^29^.

### Epithelial cells adhesion assay

*S. salivarius* strains were tested for their adhesion to hTERT-immortalized gingival keratinocytes (TIGKs, CRL-3397, ATCC). Bacterial cells included in this assay were *S. mutans* UA159 (negative control), *S. salivarius* M18 (test control), *S. salivarius* SALI-10, *S. salivarius* SALI-11, and *S. salivarius* SALI-50. TIGKs cells were cultured in a T75 Flask using Dermal Cell Basal Medium (ATCC PCS-200-030) supplemented with keratinocyte growth kit (ATCC PCS-200-040) and pen-strep (50 I.U./mL penicillin and 50 µg/mL streptomycin) until they reached 80–90% confluency. Monolayers of the TIGKs cells were formed overnight in 96-well plates. *S. salivarius* strains were grown in TSB overnight at 37 °C in 5% CO_2_ in air and then washed three times in PBS before resuspending to a final concentration of 3 × 10^9 ^c.f.u/mL in the same media used to grow TIGKs cells. TIGKs cells were washed three times with PBS before 100 µL of each bacterial cell suspension was added to TIGKs cells, followed by incubation for 2 h under the above mentioned conditions. After bacterial cells were removed, the TIGKs cells were washed three times in PBS before being dissociated from the wells by adding 30 µL of a 0.5 g/L trypsin and 0.2 g/L EDTA solution to each well. The cells were then incubated for 30 min under the same conditions mentioned above. Serial dilutions of the trypsinized cells were performed in PBS before plating on TSA plates. Plates were incubated overnight at 37 °C before CFU counting.

### Comparative genomics analysis

A comparative genomic analysis of *S. salivarius* SALI-10 and other *S. salivarius* strains was carried out using the BV-BRC. Complete genomes were downloaded in FASTA format from the BV-BRC database, and their proteomes were subsequently extracted. Homologous proteins were grouped with BV-BRC’s clustering tools, which utilize algorithms like BLASTp and OrthoMCL to form orthologous clusters. Pan-genome analysis then sorted proteins into core, accessory, and strain-specific categories. Functional annotations, such as Gene Ontology (GO) terms, metabolic pathways, and virulence factors, were assigned through BV-BRC’s pipelines. The findings were visualized using phylogenetic trees, protein similarity maps, and heatmaps, illustrating evolutionary relationships and protein distributions. Notably, unique proteins in SALI-10 were identified that could contribute to its probiotic capabilities.

### Detection of pLANs and other lantibiotics using UPLC-QTOF MS

*S. salivarius* strains were cultured in appropriate media supplemented with different types of carbon sources and incubated aerobically for 24 h at 37 °C. The CFS was separated from the cell pellets by centrifugation and mixed with an equal amount of chloroform, stirring overnight at 4 °C. The white precipitate was collected by centrifugation and dried at 38 °C for 24 h. Dried peptides (semi-purified) were resuspended in water and analyzed by UPLC-QTOF MS as described previously^18^. For further purification, semi-purified peptides were applied to C18 SEP Pack cartridges (Waters) that had been prewashed with 5 column volumes of 95% methanol containing 0.1% TFA, followed by 5 column volumes of water. The column was then washed with increasing concentrations of methanol, after which pLANs were eluted with 95% methanol supplemented with 0.1% TFA. The collected fractions were dried using a vacuum concentrator, and the dried material was resuspended in PBS to a final concentration of 1 mg/mL. Pierce™ BCA Protein Assay Kit (Thermo Scientific) was used for protein quantification.

### Sorbitol utilization assay

*S. salivarius* strains were initially grown overnight at 37 °C in TSYE containing glucose as the primary carbon source. The overnight culture was then centrifuged at 5000 × *g* for 10 min, and the cells were washed twice with PBS to remove any residual glucose. The washed cells were resuspended in minimal medium lacking sugar and adjusted to an *A*_600_ of 0.5. To assess sorbitol utilization, 20 µL of the cell suspension was inoculated into 96-well plates containing 180 µL of minimal medium supplemented with 0.5% sorbitol. A control was also set up with the same medium but without sorbitol. The cultures were incubated at 37 °C with shaking at 150 rpm. The growth of *S. salivarius* was monitored by measuring the *A*_600_ at regular intervals (every 1 h) over a 24 h period using a microplate reader. An increase in *A*_600_ in the sorbitol-containing medium relative to the control indicated that the strain could utilize sorbitol as a carbon source. Additionally, the final *A*_600_ after 24 h was compared between the test and control samples to quantify the extent of sorbitol utilization.

### Transcript-level analysis

*S. salivarius* was grown on Columbia Blood Agar for 24 h before single colonies were used to inoculate minimal medium supplemented with either 0.5% glucose or 0.5% sorbitol. The cultures were grown overnight to the indicated *A*_600_. RNA isolation and purification were performed using a combination of TRIzol Reagent (Invitrogen) and the RNeasy Mini kit (Qiagen). After quality control analysis, cDNA was synthesized from the purified RNA using the Superscript IV VILO with ezDNase Master Mix (Invitrogen), and quantitative reverse transcription polymerase chain reaction (qRT-PCR) was performed on the QuantoStudio 3 (Applied Biosystems) using PowerUp SYBR Green Master Mix (Applied Biosystems). Control samples for each condition were generated using the provided Superscript IV VILO master mix, which lacked the reverse transcriptase enzyme, and were included in all qRT-PCR experiments. Comparison of transcript levels was performed using the *∆*CT method, with *16S rRNA* as the endogenous control gene. The primers used for qRT-PCR are listed in Table S5.

### Promoter region analysis of *srnA*_1-4_

Promoter regions upstream of *srnA*_1-4_ on the minus strand were characterized based on canonical bacterial promoter motifs using SnapGene (v8.1.0). Each area was evaluated for the presence and spacing of the CRP-binding site, the −35 and −10 boxes, Shine-Dalgarno (SD) sequences, and translational start codons. Promoters were annotated with respect to sequence consensus: TGTGAN6TCACA, TTGACA, TATAAT, optimal motif spacing: 16–18 bp for the −35 and −10, ~100 bp upstream for the CRP binding site from the start of the *srnA*_1-4_ genes, and SD (GGAG) position (typically 6–10 bp upstream of the start codon).

### *S. salivarius* SALI-10 transplantation to the human oral cavity

This open-label, single-arm, 7-day exploratory pilot study evaluated the short-term effects of *S. salivarius* SALI-10 lozenges on the oral microbiome and oPMN counts in healthy adults. We created easy-to-dissolve oral lozenges containing ~1 × 10¹⁰ CFU of *S. salivarius* SALI-10 and used them as the delivery system for oral cavity transplantation. The intervention protocol was approved by Veritas IRB (Protocol #2025-3676-20642-6) and conducted in accordance with the Declaration of Helsinki. The trial is registered at ClinicalTrials.gov (Identifier: NCT06819761). After telephone screening and electronic informed consent, eight participants received seven lozenges and two oral microbiome and oral rinse collection kits. Participants collected baseline (Day 0, pre-transplantation) oral microbiome and oral rinse samples at home, then self-administered one lozenge nightly after routine toothbrushing for 7 days. Twenty-four hours after the final dose (Day 7, post-transplantation), participants collected a second set of oral microbiome and oral rinse samples. Oral microbiome samples underwent DNA extraction and qPCR to measure changes in the relative abundance of periodontal pathogens (*P. gingivalis, T. forsythia, T. denticola, P. micra*, and *F. nucleatum*) and the copy number of the *srnA2* gene (a marker for pLANs population). Oral rinse samples were analyzed for oPMN counts as a surrogate marker of OIL. All samples were de-identified and destroyed immediately after analysis. Paired baseline-to-Day-7 changes were assessed using the Wilcoxon signed-rank test, with significance defined as *p* < 0.05. Participants were monitored for adverse events, with mild transient oral irritation considered the primary expected effect.

### Quantification of the oPMN counts

Five hundred microliters of oral rinse samples were centrifuged at 4000 rpm to pellet the cellular fraction. The cell pellets were resuspended in 100 µL PBS and stained with 1 µL of Acridine Orange (Invitrogen) to label polymorphonuclear neutrophils (PMNs) selectively. Ten-microliter aliquots of the stained cell suspension were loaded onto a Neubauer hemocytometer and examined under a fluorescence microscope. Neutrophils (identified by characteristic nuclear fluorescence) were counted in multiple grid fields, and the concentration of neutrophils in each sample was calculated and expressed as cells per milliliter of oral rinse, representing the oral inflammatory load^76^.

### In silico assessment of the *srnA*2 qPCR primer specificity

Primer specificity was assessed in silico using NCBI Primer-BLAST. The forward and reverse primers targeting *srnA2* were queried against the NCBI nucleotide (nt) database. Up to 50,000 target sequences were permitted, and 33,106 BLAST hits were analyzed. Primer binding required ≥2 total mismatches, including ≥2 mismatches within the 3′ terminal 5 nucleotides; targets with ≥6 mismatches were excluded. Predicted PCR products were constrained to 61–220 bp (maximum target size 220 bp). Primer length was limited to 15–25 nt (optimal 20 nt), melting temperatures to 55–63 °C (optimal 58 °C), with a maximum forward-reverse Tm difference of 3 °C; repeat masking and low-complexity filtering were enabled. Searches were performed with a BLAST word size of 7 and an E-value threshold of 30,000. Predicted amplification products were compiled into a summary table to distinguish exact on-target matches from closely related homologous loci. Under these stringency settings, predicted products were primarily identified for *S. salivarius srnA2* sequences, and a number of predicted products were also returned for homologous loci annotated in select *S. pneumoniae* genomes (Table S6). As discussed previously^18^, *S. pneumoniae* does not produce intact pLANs from these loci. Because in silico analyses are limited by available reference sequences, qPCR results are interpreted as enrichment of an *srnA2*-harboring population rather than definitive strain-level identification.

## Supplementary information

Supplementary materials.

## Data Availability

All data supporting this study are provided in the main text and supplementary materials. Raw subgingival metagenomic sequencing data are available in NCBI BioProject PRJNA1295854, and raw multispecies biofilm metagenomic sequencing data are available in NCBI BioProject PRJNA1142934 and PRJNA1311577.
