# Supplementary material for: Phosphorylated lantibiotics-producing commensals integrate into the human oral microbiome to suppress pathogens and promote microbiome homeostasis
Source: NPJ Biofilms Microbiomes. 2026 Apr 3;12:101. doi: 10.1038/s41522-026-00976-y (PMC13219514; doi:10.1038/s41522-026-00976-y)
Supplement: Supplementary file 1 — Supplementary materials. [file 41522_2026_976_MOESM1_ESM.docx]

Supplementary Materials for

**Phosphorylated lantibiotics-producing commensals integrate into the human oral microbiome to suppress pathogens and promote microbiome homeostasis.**

Abdelahhad Barbour *et al.*

*Corresponding author. Email: [abdelahhad.barbour@utoronto.ca](mailto:abdelahhad.barbour@utoronto.ca)

**This PDF file includes:**

Supplementary Text

Figs. S1 to S7

Tables S1 to S6

Supplementary Text

**Adhesion Strategies of pLANs-producing *S. salivarius* SALI-10: Roles of CshA, SRR Glycoproteins, and Adhesin P1**

Considering their potential role in adhesion and colonization, we focused on eight secreted proteins that feature Gram-positive cell surface anchor motifs, including CshA fibrils, serine-rich glycoproteins, and Adhesin P1. All of these have been previously shown to provide key advantages in adhesion and colonization.

***Pili, fimbriae, and fibrils***

A key contributor to S. salivarius SALI-10’s adhesion capability is a fibrillar adhesin-related protein containing 24 CshA/Fibril repeats (PFAM19076, TIGR04225) (Fig. S4A). CshA plays a crucial role in bacterial auto-aggregation, epithelial adherence, and biofilm cohesion, allowing S. salivarius to integrate into polymicrobial communities (*1*). CshA facilitates stable bacterial attachment under shear forces, possibly contributing to biofilm stabilization​ (*2*). Notably, deletion studies confirm its essential function in maintaining biofilm stability (*1*). CshA expression varies across S. salivarius strains, with only 6 out of 10 analyzed genomes encoding this adhesin. Strains lacking CshA-like proteins, such as M18, CCHSS3, and ATCC 25975, may rely on alternative adhesion strategies, suggesting evolutionary divergence based on environmental pressures (Fig. S2B). Sequence similarity network (SSN) analysis of CshA revealed a distinct phylogenetic cluster comprising S. salivarius and S. parasanguinis, while S. mitis and S. gordonii formed separate clusters (Fig. S4C).

***Serine-rich repeat (SRR) glycoproteins***

SRR glycoproteins, including SrpA, SrpB, and SrpC, are critical mediators of host adhesion and biofilm establishment and are secreted via the secA2/Y2 system, which plays a vital role in S. salivarius epithelial colonization (*3*). SALI-10 demonstrated a robust capacity to adhere to gingival epithelial cells, with genomic analysis confirming a complete secA2/Y2 locus encoding the three SRR glycoproteins, similar to S. salivarius JIM8777 (Fig. S4D). However, strain K12, while maintaining the secA2/Y2 system, lacked srpABC genes, relying on a single distally encoded SRR protein, resembling SrpC from SALI-10 and JIM7888. Strain M18 exhibited a modified secA2/Y2 locus with different genetic organization and devoid of SRR glycoproteins, while CCHSS3 harbored three SRR proteins with lower sequence similarity (Fig. S4D).

Given the absence or divergence of SrpA in strains K12, M18, and CCHSS3, we employed SWISS-MODEL to predict its structural organization in SALI-10. The gene encoding the SrpA protein (2,208 amino acids) was identified between 870,607 and 877,038 bp on the complementary strand. BLAST analysis revealed significant sequence similarity to fimbrial adhesins from S. pneumoniae (PsrP, 59.4%), S. gordonii (GspB, 57%), and S. parasanguinis (Fap1, 51.3%). Structural modeling highlighted a unique domain (SALI-10: SrpA319-487) with 64.84% identity to the KRT10-binding region of PsrP, suggesting its involvement in epithelial adherence. This domain is followed by an extended serine-repeat region and a classic LPxTG cell wall sorting motif, crucial for peptidoglycan anchoring by sortases (Fig. S4E, F).

***Csp operon and Adhesin P1***

Antigen I/II (AgI/II, also known as CspB) is a multifunctional adhesin mediating interactions with salivary pellicle components and host mucosal surfaces (*4, 5*). Comparative genomics revealed an intact csp operon in SALI-10, K12, M18, and YU10. SALI-10 Adhesin P1 comprises a 42-residue signal sequence, an uncharacterized N-terminal region, three alanine-rich repeats (A1-3), a central variable region (V), three proline-rich repeats (P1-3), and a C-terminal LPxTG motif (Fig. S4G, H). Unlike S. mutans, the AgI/II (CspB) protein in SALI-10 lacks the C3 region (Fig. S4H). The presence of this adhesin within an intact csp operon suggests a fundamental role in fimbriae formation and host adaptation.

The diverse array of cell wall-anchored adhesins in S. salivarius SALI-10 highlights its evolutionary adaptation to the oral microbiome. By leveraging multiple adhesion strategies, including CshA-mediated biofilm stabilization, SRR glycoprotein secretion via the secA2/Y2 system, and AgI/II-mediated host interactions, SALI-10 exhibits remarkable potential for niche adaptability.

**Supplementary Methods**

### ****Screening for antimicrobial peptides produced by**** S. salivarius

Single colonies of each of the eighty S. salivarius isolates were used to inoculate 5 mL of TSYE broth and incubated overnight at 37 °C under 5% CO_2_. Overnight cultures were adjusted to an *A*_600_ of 0.8 prior to plating. Equal volumes of each standardized culture were seeded onto the surface of TSYECa soft agar plates (final agar concentration 0.8%), with 20 mL of this medium per plate. For each isolate, 20 plates were prepared and incubated overnight under the conditions described above. Following incubation, plates were frozen at -80°C overnight and subsequently thawed at 70°C for 30 min to release AMPs-containing liquid. The resulting liquid material was clarified by centrifugation at 18,000 × *g* for 60 min. For each isolate, 250 mL of clarified supernatant was mixed with an equal volume of chloroform and stirred overnight at 4°C. Samples were then centrifuged at 18,000 × *g* for 60 min, after which both the aqueous and chloroform phases were discarded. The white interfacial precipitate was collected and dried overnight at 38°C. Dried material was resuspended in 5 mL of 35% acetonitrile containing 0.1% trifluoroacetic acid and subjected to SPE using C18 cartridges pre-conditioned with 8 bed volumes of methanol followed by water. Columns were sequentially washed with 8 bed volumes each of 10%, 30%, 50%, and 80% methanol. Final elution was performed using 8 bed volumes of 95% methanol containing 0.1% TFA. All eluates were dried using a SpeedVac concentrator and resuspended in PBS. Protein concentration of all fractions was normalized to 1 mg/mL stocks. For screening assays, final concentration of 100 µg of each normalized fraction was applied to each well in well-diffusion assays, as described in the Methods section in the main manuscript. Under the assay conditions used, fractions eluted with up to 80% methanol did not exhibit detectable antimicrobial activity. In contrast, fractions eluted with 95% methanol retained the majority of antibacterial activity and were therefore used for comparative screening of AMPs production across S. salivarius isolates.

To further validate that the extraction and purification workflow was not biased toward pLANs and was broadly suitable for isolating S. salivarius lantibiotics, we applied the same production and extraction pipeline to S. salivarius strain K12, a well-characterized producer of the non-phosphorylated lantibiotics salivaricin A and salivaricin B. Using this identical agar-based growth, freeze–thaw extraction, chloroform precipitation, and C18 solid-phase extraction workflow, both salivaricin A and salivaricin B were successfully detected by high-resolution mass spectrometry at the expected molecular masses (Fig. S3). These results confirm that the extraction protocol reproducibly captures both phosphorylated and non-phosphorylated salivaricins and is robust to strain-to-strain differences in growth or lantibiotic chemistry.


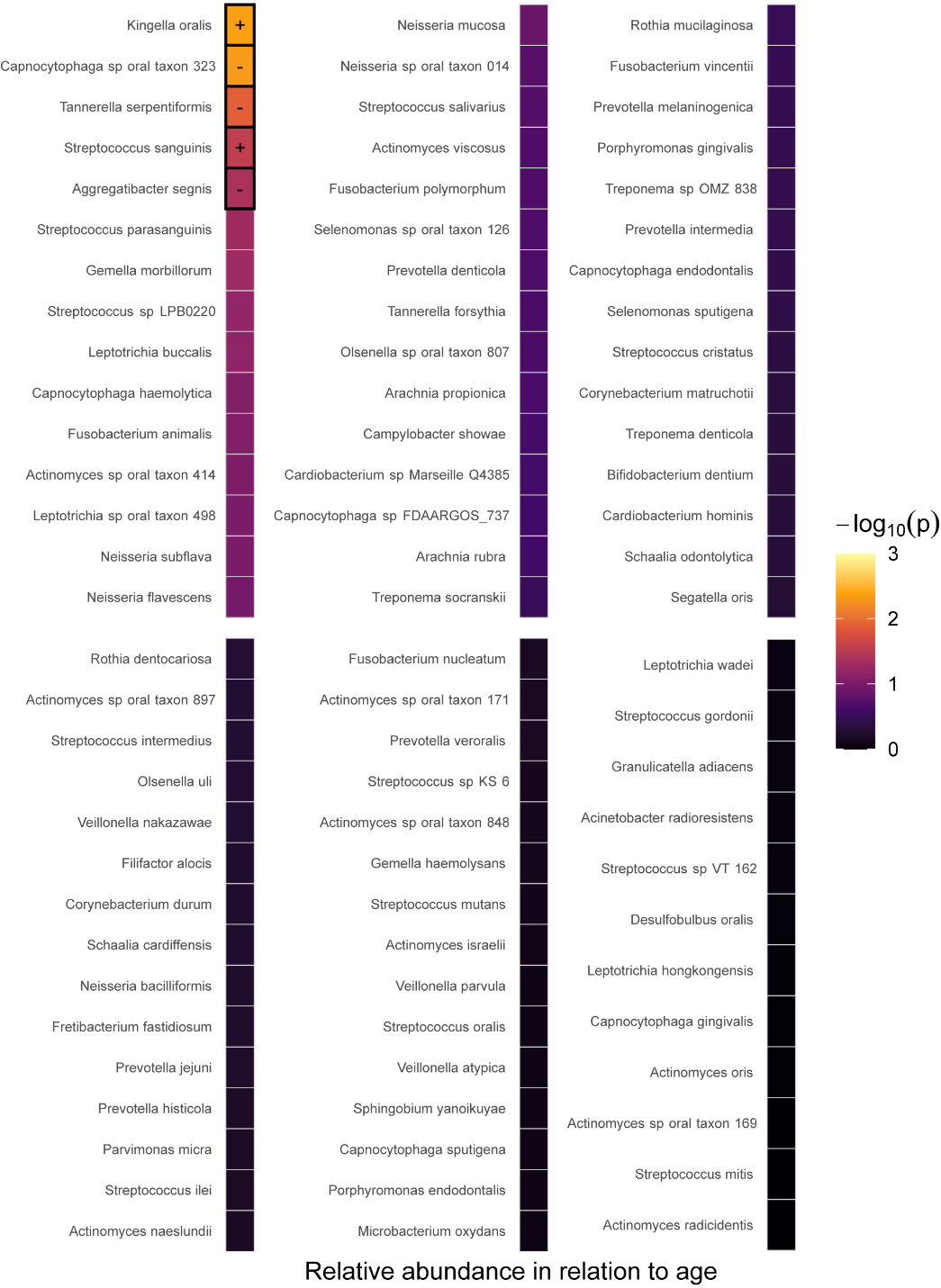


**Fig. S1. Age-associated microbial taxa identified by MaAsLin2.** Heatmap summarizing taxa associated with age based on MaAsLin2 models, including age as a fixed effect. Tile color represents statistical significance expressed as −log10(p), with warmer colors indicating stronger associations. Taxa with significant associations (*p* < 0.05) are outlined in black, and the direction of the association with age is indicated for significant features only (“+” indicates increasing abundance with age, “−” indicates decreasing abundance with age).


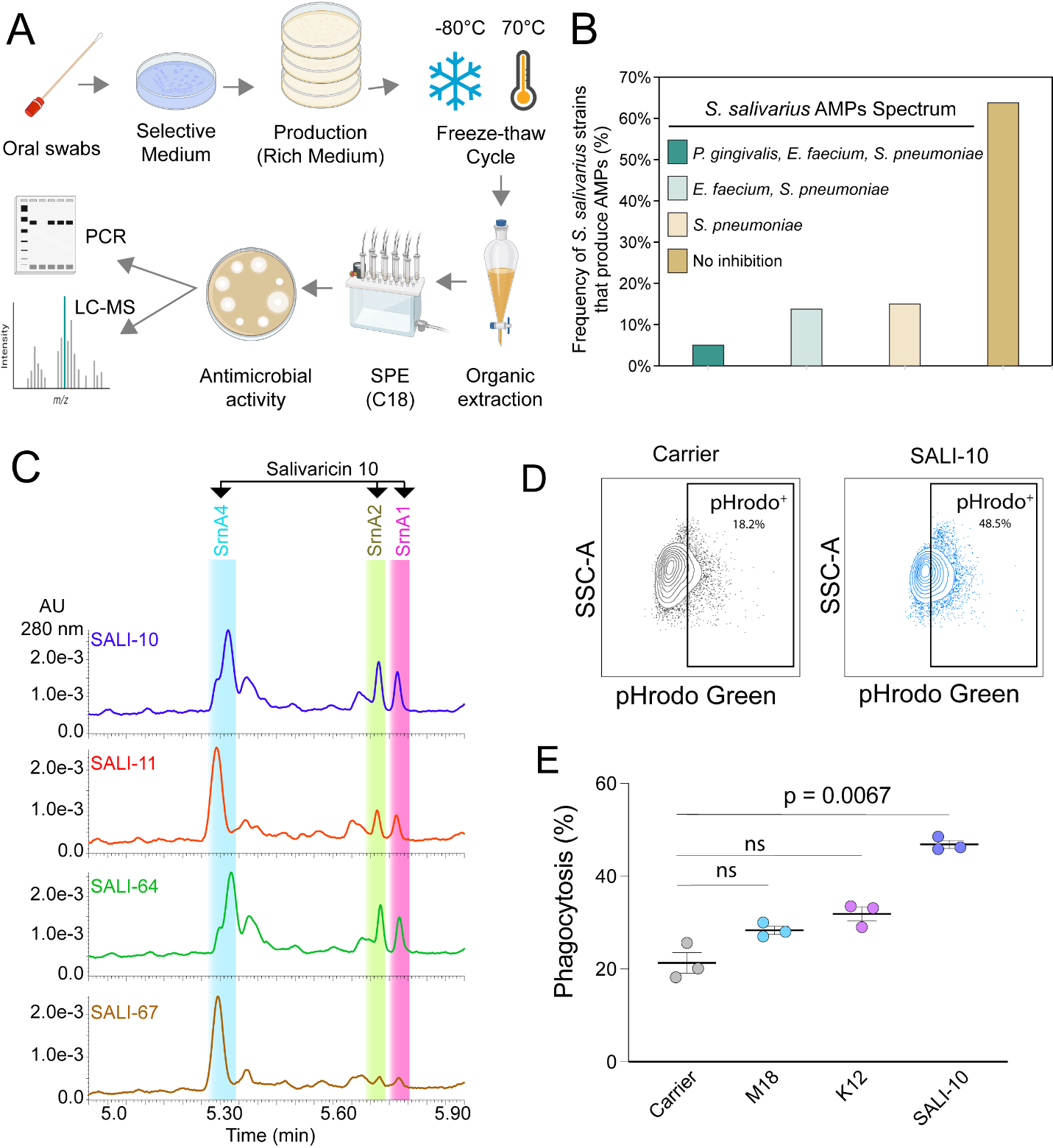


Fig. S2. Phosphorylated lantibiotics from *S. salivarius* exhibit enhanced antimicrobial and pro-immune activity despite their lower prevalence among oral isolates. (A) Schematic overview of the AMP extraction and screening pipeline used to identify bioactive peptides from *S. salivarius isolates* (n = 80) collected from healthy oral microbiomes. (B) Distribution of antimicrobial activity across isolates, showing the proportion of strains producing AMPs effective against *P. gingivalis*, MDR *S. pneumoniae*, and VRE. Only 5% of strains inhibited all three pathogens, while 65% showed no detectable AMP activity. All AMPs fractions were adjusted to a final concentration of 1 mg/mL before 100 µg was used in well-diffusion assays (methods section). (C) UPLC-QTOF-MS analysis showing that all of the four *S. salivarius* strains (SALI-10, SALI-11, SALI-64 and SALI-67) which inhibited the three targeted pathogens secrete salivaricin 10 phosphorylated lantibiotics (SrnA1, SrnA2, SrnA4). (D) Representative contour plots of flow cytometry analysis for neutrophil phagocytosis *in vitro* using pHrodo-labeled *S. pneumoniae* ATCC6301. (E) Only phosphorylated lantibiotics (AMP extracts from strain SALI-10), and not other *S. salivarius*-derived AMPs (from K12 and M18 strains), enhance phagocytic uptake of pathogens. Data are presented as means ± by the Kruskal-Wallis test.

**
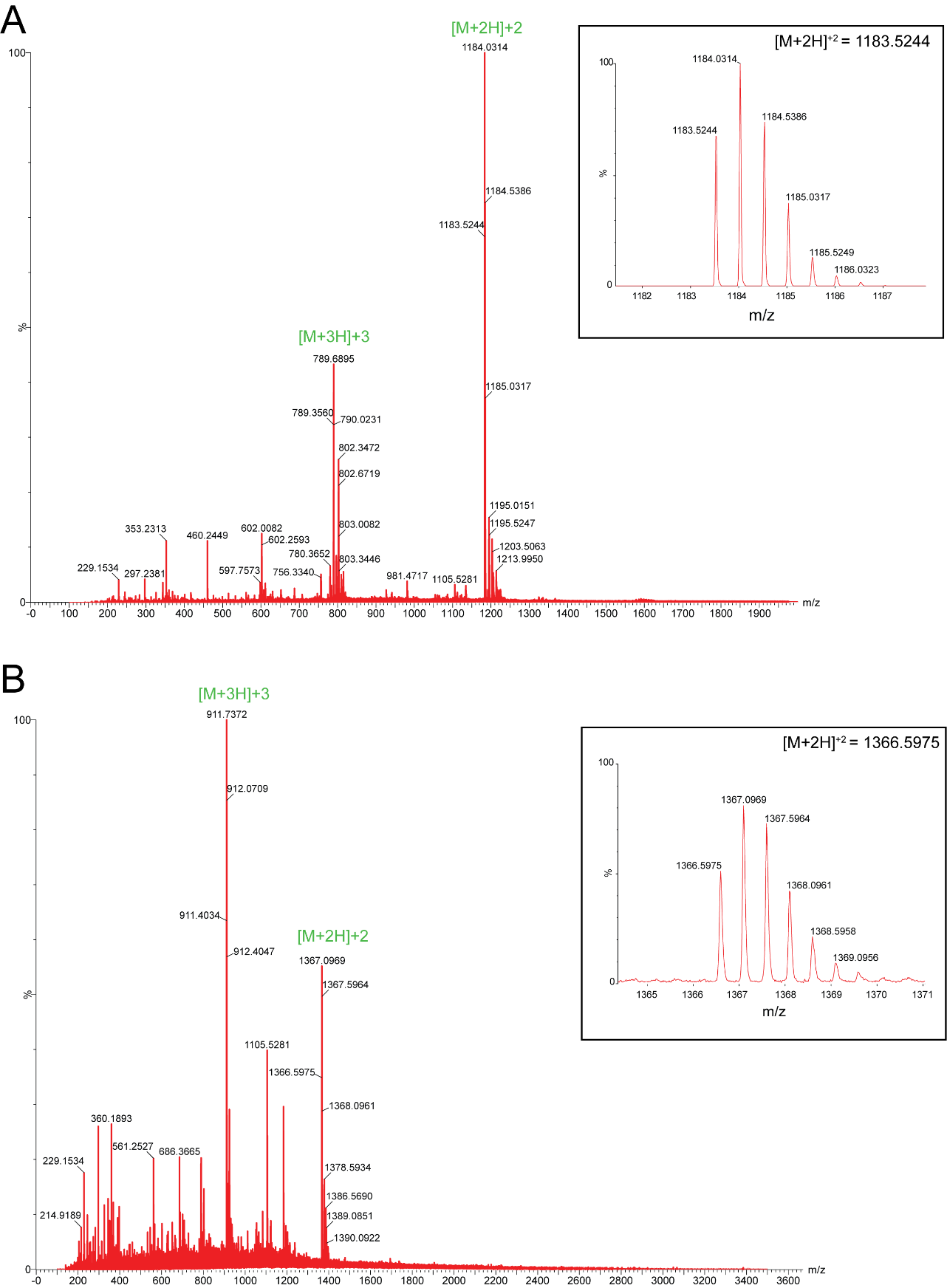
**

**Fig. S3. ESI-MS analysis of non-phosphorylated lantibiotics produced by *S. salivarius*.** In addition to the pLANs produced by SALI-10 reported in the main text, our screening method enabled isolation of non-phosphorylated lantibiotics from other producer strains, including salivaricin A2 (**A**) and salivaricin B (**B**). Inset values indicate the monoisotopic mass of the doubly charged ion for each lantibiotic.


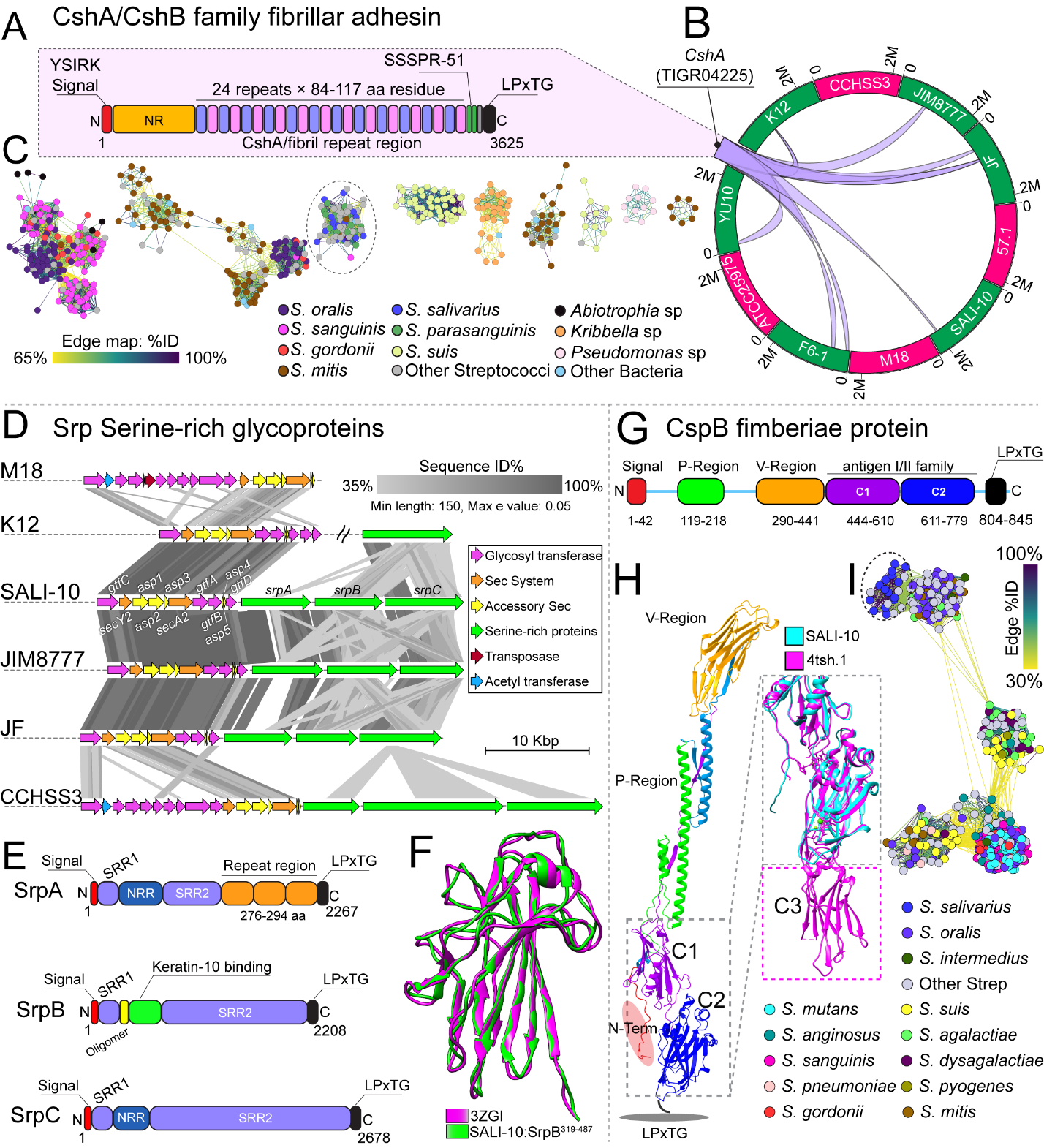


Fig. S4. Structural features of three strain-specific streptococcal cell surface systems in pLANs-producing *S. salivarius* SALI-10 involved in adherence and colonization. (A) The SALI-10 fibrillar adhesin CshA includes an N-terminal YSIRK signal peptide (red), a non-repetitive region (orange), 24 repeats (pink/purple), an SSSPR-51 domain (green), and a C-terminal LPXTG motif (black). (B) CshA is found in 6 out of 10 *S. salivarius* genomes analyzed via BLAST. (C) Sequence similarity network (SSN) of CshA in different bacteria. Nodes represent protein sequences; edges represent BLASTP hits. The *S. salivarius* group clusters with *S. parasanguinis* (65% ID cut-off, dashed circle). (D) Comparative genomic analysis of the secA2/Y2 locus encoding serine-rich glycoproteins in six *S. salivarius* strains. SALI-10 and JIM7888 show auto-aggregated phenotypes and intact loci; K12 has a non-auto-aggregated phenotype with one SRR at a distant genomic location; M18 shows a different locus organization with no SRR. (E) Schematic of SrpA, SrpB, and SrpC structures in SALI-10. SrpA has five domains: signaling sequence, SRR1, Keratin-10 binding region, SRR2, and LPxTG cell wall region. (F) Structure simulation of the KRT10-binding region of SALI-10 SrpA superimposed with PsrP protein (PDB 3ZGI). (G) Domain structure of CspB/Adhesin_P1 fimbriae associated protein inferred from SALI-10 genomic data. Key domains: Adhesin_P1_N (pfam18652, P-region), BspA variable domain (pfam18220, V-region), and antigen I/II family C terminus (NF033804, C1 and C2). (H) Proposed tertiary model of SALI-10_P1 by AlphaFold, highlighting the antigen I/II family region (C1 and C2) superimposed with *S. mutans* Ag I/II (4tsh), with C3 region absent in *S. salivarius_*P1. (I) Protein similarity map of Adhesin_P1 in Streptococcus. Nodes represent protein sequences; edges represent sequences with alignment score cut-off 120 and length 600-1600 aa. *S. salivarius* group highlighted (dashed circle).


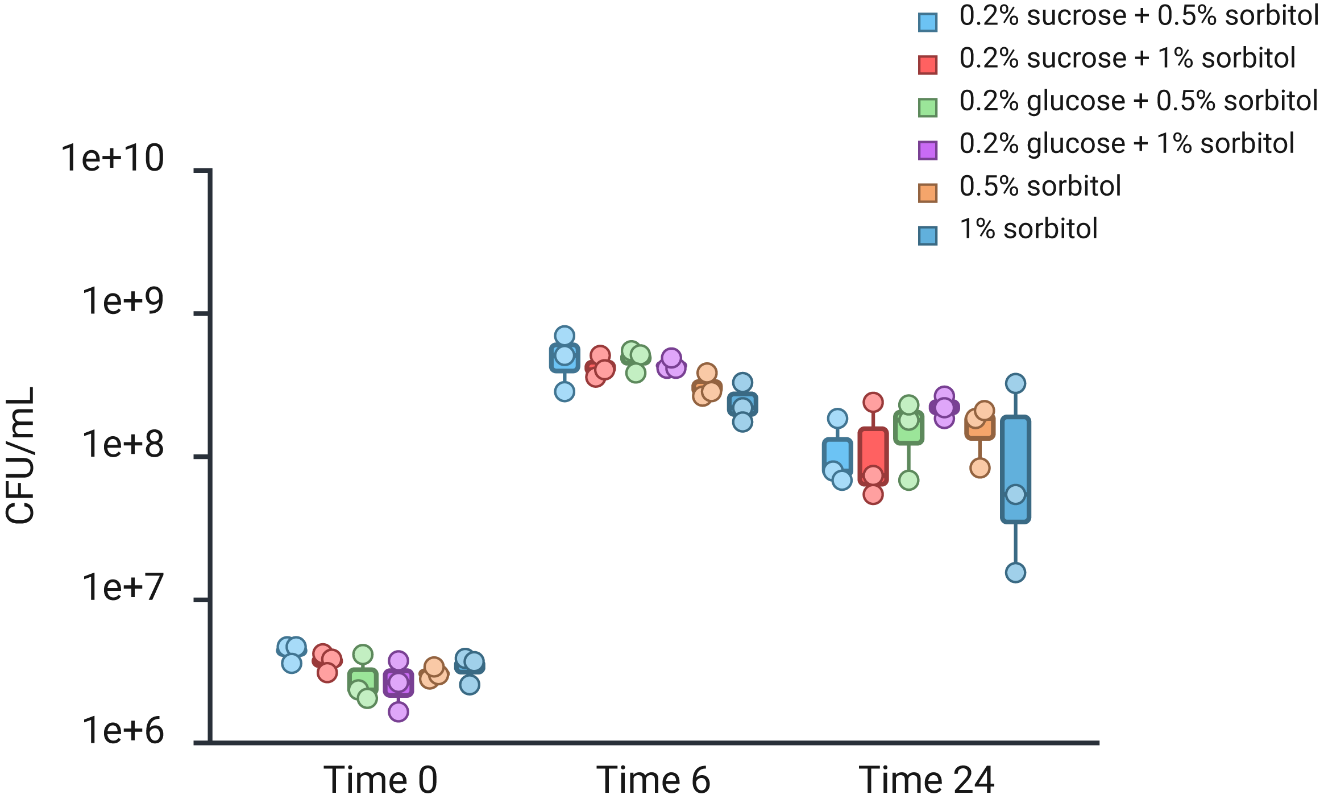


Fig. S5. Colony-forming units of *S. salivarius* SALI-10 grown with different carbon source compositions over time.


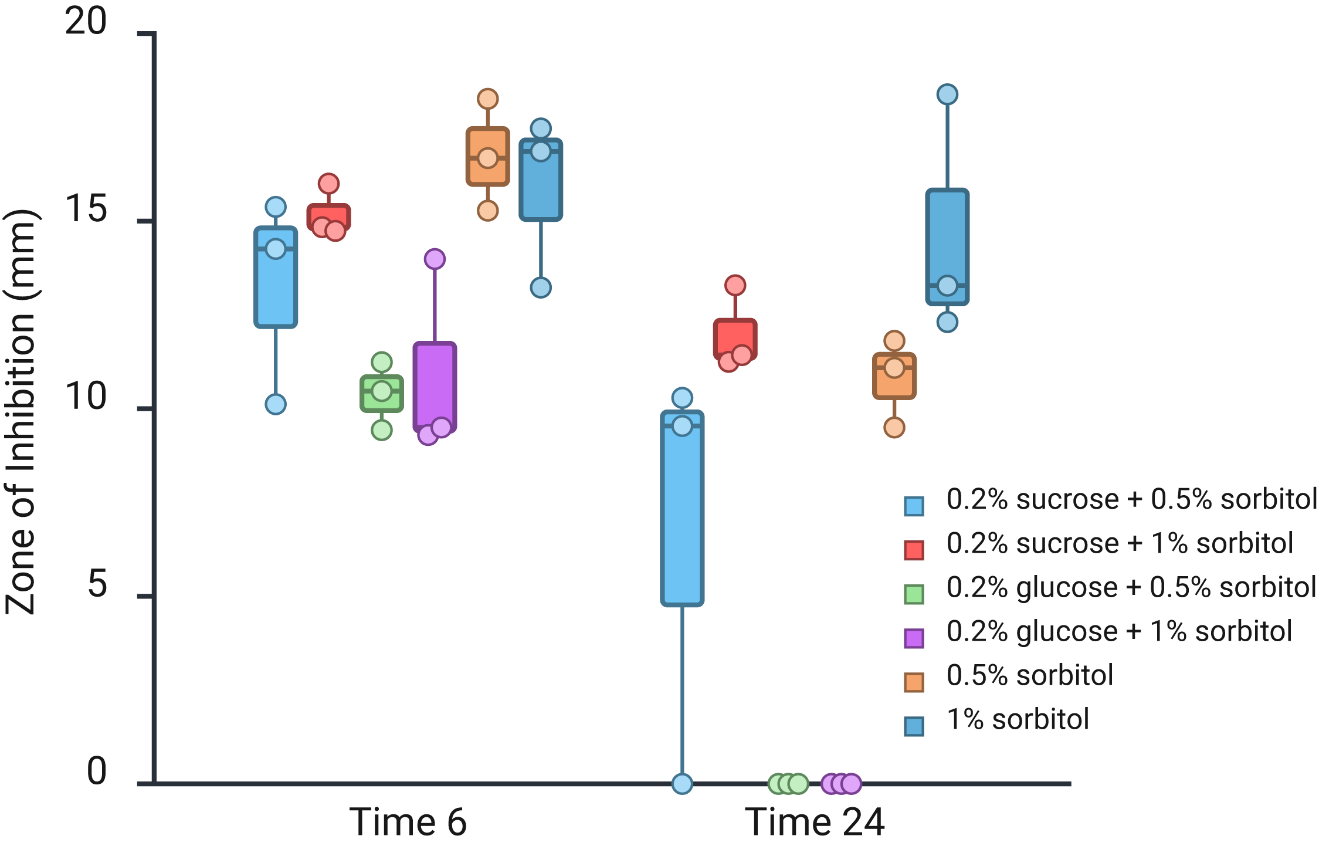


Fig. S6. Inhibitory activity of cell-free supernatants (CFS) from *S. salivarius* SALI-10 grown with different carbon sources over time, measured by zone of inhibition diameter using a well diffusion assay.


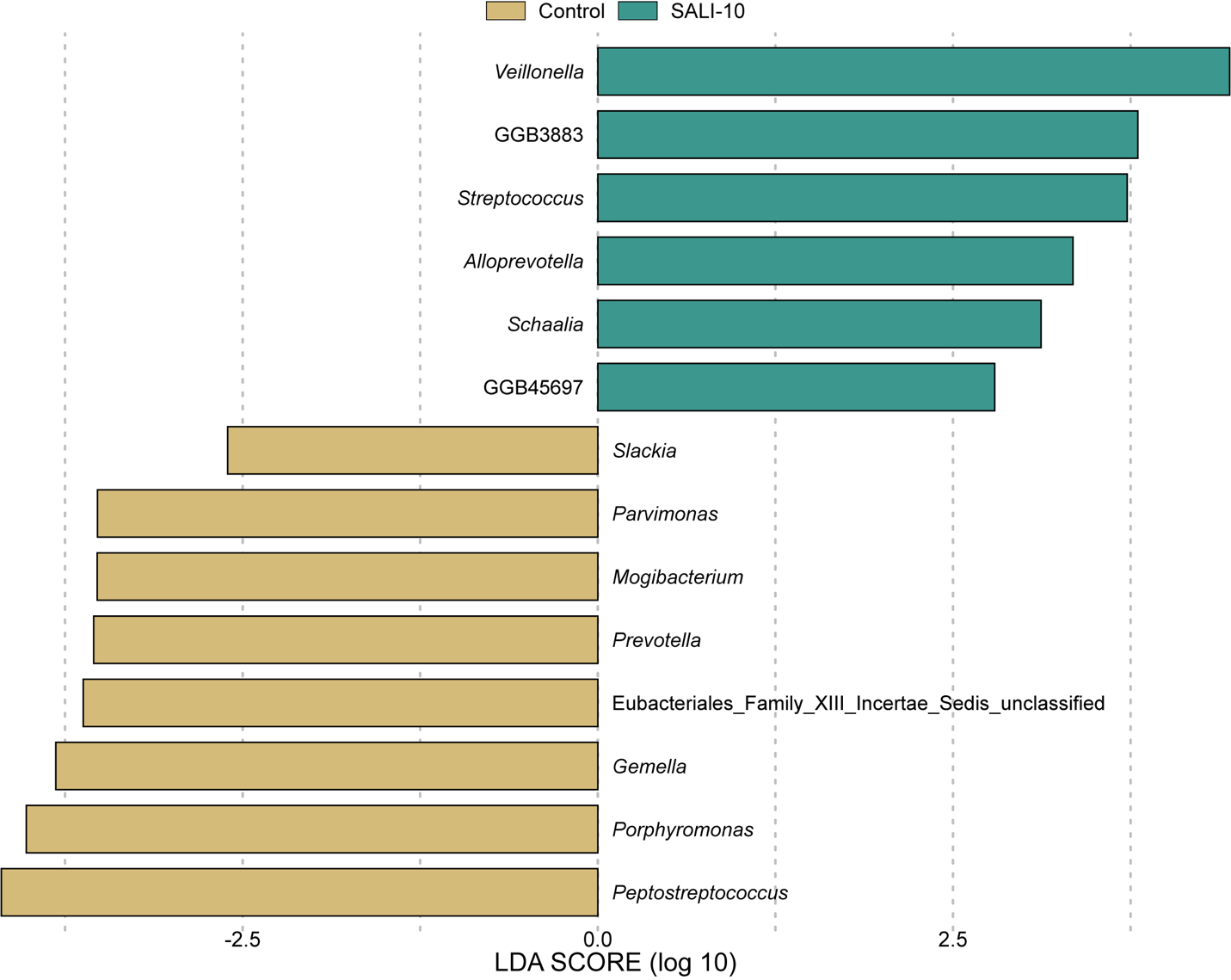


**Fig. S7.** Linear discriminant analysis (LDA) of bacterial genera significantly reduced or enriched by *S. salivarius* SALI-10 treatment in a dysbiotic multispecies biofilm.

Table S1. Demographic and Clinical Characteristics of Study Participants

| **ID** | **Group** | **Age (years)** | **Sex** | **No. of Teeth** | **GI (sampled)** | **GI (overall)** | **PD (mm)** | **Stage** | **Grade** |
| --- | --- | --- | --- | --- | --- | --- | --- | --- | --- |
| 1 | Healthy | 62 | F | 23 | – | – | – | – | – |
| 2 | Healthy | 31 | M | 32 | – | – | – | – | – |
| 3 | Healthy | 29 | F | 28 | – | – | – | – | – |
| 4 | Healthy | 66 | M | 28 | – | – | – | – | – |
| 5 | Healthy | 29 | M | 28 | – | – | – | – | – |
| 6 | Healthy | 25 | F | 28 | – | – | – | – | – |
| 7 | Healthy | 47 | F | 28 | – | – | – | – | – |
| 8 | Healthy | 23 | M | 28 | – | – | – | – | – |
| 9 | Healthy | 62 | M | 28 | – | – | – | – | – |
| 10 | Healthy | 62 | F | 28 | – | – | – | – | – |
|  | **Mean ± SD** | 43.60 ± 17.90 | – | 24.10 ± 4.33 | – | – | – | – | – |
| 11 | Periodontitis | 67 | M | 23 | 2.30 | 1.98 | 7 | IV | C |
| 12 | Periodontitis | 74 | M | 23 | 1.83 | 1.32 | 7 | IV | B |
| 13 | Periodontitis | 58 | M | 21 | 2.00 | 1.96 | 9 | IV | C |
| 14 | Periodontitis | 76 | M | 20 | 1.83 | 1.94 | 8 | IV | C |
| 15 | Periodontitis | 61 | F | 23 | 2.00 | 2.00 | 5 | IV | C |
| 16 | Periodontitis | 39 | M | 32 | 1.83 | 1.50 | 8 | III | C |
| 17 | Periodontitis | 70 | F | 18 | 1.83 | 1.69 | 8 | III | B |
| 18 | Periodontitis | 58 | M | 24 | 2.00 | 1.53 | 7 | III | C |
| 19 | Periodontitis | 55 | M | 29 | 1.67 | 1.47 | 8 | IV | C |
| 20 | Periodontitis | 53 | F | 28 | 1.00 | 1.09 | 6 | III | B |
|  | **Mean ± SD** | 61.10 ± 11.10 | – | 27.90 ± 2.13 | 1.829 ± 0.337 | 1.648 ± 0.317 | 7.30 ± 1.16 | – | – |
|  | ***p*-value** | **0.0171*** | – | **0.0228*** | – | – | – | – | – |

^PD, probing depth; GI, Gingival Index.^

^Demographic data are presented as mean ± standard deviation (SD); group comparisons for continuous variables were performed using^ **^chi-squared tests.^**

Table S2. Biological features of phosphorylated lantibiotic-producing *S. salivarius* strains

| **Strain** | **Salivaricin 10 BGC^Ω^** | | | | | **Adhesion genes^Ω^** | | | **Sorbitol^§^** | **Antibiotic Resistance^‡^** | |
| --- | --- | --- | --- | --- | --- | --- | --- | --- | --- | --- | --- |
|  | *srnA1* | *srnA2* | *srnA4* | *srnM* | *srnT* | *CshA* | *SrpA* | *Adhesin P1* |  | Ery | Tet |
| SALI-10 | + | + | + | + | + | + | + | + | Yes | S | S |
| SALI-11 | + | + | + | + | + | + | + | + | Yes | R | S |
| SALI-64 | + | + | + | + | + | + | + | + | Yes | R | R |
| SALI-67 | + | + | + | + | + | + | + | + | Yes | R | R |
| K12 | - | - | - | - | - | + | - | + | No | S | S |
| M18 | - | - | - | - | - | - | - | + | No | S | S |

**^Ω^**Genes presence or absence assay was performed using PCR, (+) = presence of the gene, (-) = absence of the gene.

**^§^**Metabolic profile and fermentation of sorbitol was done using API STRP 20 kit, (Yes) = the strain can ferment sorbitol, (No) = the strain can not ferment sorbitol.

**^‡^** Antibiotic susceptibility test performed using Disc diffusion Assay following CLSI guidelines. Ery = Erythromycin, Tet = Tetracycline. (R) = The strain is resistant to this antibiotic, (S) = the strain is sensitive to this antibiotic

**Table S3.** Purification of pLANs using Chloroform extraction and C18-Solid Phase Extraction.

| Step | Activity  (AU/ml) | Total Activity (AU) | Total Protein  (mg) | Specific activity  (AU/mg) | Yield  (%) | Purification  (fold) |
| --- | --- | --- | --- | --- | --- | --- |
| CFS^§^ | 200 | 200,000 | 10,500 | 19 | 100 | 1 |
| CHCl_3_ | 6,400 | 147,200 | 389.5 | 378 | 74 | 20 |
| C18-SPE | 25,600 | 115,200 | 3.2 | 36,000 | 58 | 1,890 |

^§^Cell-free supernatant from sorbitol culture.
AU: Antimicrobial arbitrary units.

Table S4. Indicator strains and growth conditions used in the study

| **Species** | **Strain** | **Original isolation source** | **Culture source** | **Growth medium** | **Growth conditions (atmosphere, hr)** | **Figures** | **Antibiotic resistance profile** |
| --- | --- | --- | --- | --- | --- | --- | --- |
| *Actinomyces naeslundii* | ATCC 19039 | Plaque from a tooth | ATCC^§^ | TSYB | Anaerobic, 48 h | Fig. 4 | ND |
| *Bifidobacterium dentium* | ATCC 27534 | Dental caries | ATCC | Blood-TSB | Anaerobic, 48 h | Fig. 2/3 | ND |
| *Enterococcus faecium* | W97542221 | Gut | FoD^β^ | TSYB | Aerobic, 24 h | Fig. 4/6 | VAN |
| *Fusobacterium nucleatum* | ATCC 10953 | Inflamed gingiva | ATCC | TSYB | Anaerobic, 48 h | Fig. 4 | ND |
| *Haemophilus influenzae* | Z51903604 | Pituitary tissue | MSH^¥^ | Blood-TSB | 95% air / 5% CO₂, 48 h | Fig. 4 | AMP |
| *Lacticaseibacillus rhamnosus* | ATCC 11981 | Milk products | ATCC | TSYB | 95% air / 5% CO₂, 24 h | Fig. 4 | ND |
| *Listeria monocytogenes* | U3012305 | Blood | MSH | BHI | Aerobic, 24 h | Fig. 4 | AMP |
| *Moraxella catarrhalis* | V5030446 | Blood | MSH | Blood-TSB | 95% air / 5% CO₂, 24 h | Fig. 4/6 | ND |
| *Porphyromonas gingivalis* | ATCC 33277 | Gingival sulcus | ATCC | THYEHM | Anaerobic, 72-120 h | Fig. 4/6 | ND |
| *Prevotella intermedia* | ATCC 49046 | Periodontal pocket | ATCC | THYEHM | Anaerobic, 48 h | Fig. 4/6 | ND |
| *Staphylococcus epidermidis* | CM1016 | Human breastmilk | FoD | TSB | Aerobic, 24 h | Fig. 4 | ND |
| *Streptococcus agalactiae* | W73106655 | Blood | MSH | THB | 95% air / 5% CO₂, 24 h | Fig. 4 | CLI, ERY |
| *Streptococcus anginosus* | 93B-5 | Subgingival plaque | FoD | THB | 95% air / 5% CO₂, 24 h | Fig. 4 | MTZ, AZM |
| *Streptococcus gordonii* | 76B-5 | Subgingival plaque | FoD | THB | 95% air / 5% CO₂, 24 h | Fig. 4 | MTZ, AZM |
| *Streptococcus sanguinis* | 70A-13 | Subgingival plaque | FoD | THB | 95% air / 5% CO₂, 24 h | Fig. 4 | MTZ, AZM, CLI, TE, ERY |
| *Streptococcus mitis* | 140B-8 | Subgingival plaque | FoD | THB | 95% air / 5% CO₂, 24 h | Fig. 4 | MTZ |
| *Streptococcus mutans* | UA159 | Child with active caries | ATCC | THB | 95% air / 5% CO₂, 24 h | Fig. 4/6 | ND |
| *Streptococcus pneumoniae* | W81002644 | Respiratory tract | MSH | THB | 95% air / 5% CO₂, 24 h | Fig. 4/6 | OXA, PENIV, SXT |
| *Streptococcus pyogenes* | W51503011 | Upper respiratory tract | MSH | THB | 95% air / 5% CO₂, 24 h | Fig. 4 | CLI, ERY |
| *Veillonella dispar* | ATCC 17748 | Oral cavity | ATCC | TSYB | Anaerobic, 48 h | Fig. 4 | ND |
| *Acinetobacter baumannii* | Y91300784 | Blood | MSH | TSYB | Anaerobic, 24 h | Fig. 4 | ND |

^§^American Type Culture Collection

^¥^Mount Sinai Hospital, Toronto, Canada

^β^Faculty of Dentistry, Toronto, Canada

ND = not determined.

Media abbreviations: TSYB, tryptic soy broth + 0.8% yeast extract; TSB, tryptic soy broth; THB, Todd Hewitt broth; BHI, brain heart infusion; THYEHM: Todd Hewitt broth + 0.8% Yeast extract + hemin + medadione

Antibiotic resistance abbreviations: QD, Quinupristin-Dalfopristin; TET, Tetracycline; ERY, Erythromycin; VAN, Vancomycin; AMP, Ampicillin; CLI, Clindamycin; AZM, Azithromycin; MTZ, Metronidazole; TE, Tetracycline; OXA, oxacillin; PENIV, penicillin V; SXT, Trimethoprim/Sulfamethoxazole.

Table S5. Primers used in this study

| **Gene/bacteria** | **Primer sequence 5’−3’** | **References** |
| --- | --- | --- |
| *Detection and quantification of salivaricin 10 BGC genes* | | |
| *srnA1* | AGG AAG GAG GCA ACC TTA TG | (*6*) |
|  | AGC ATC GAA ATC TAG GCT ATC |  |
| *srnA2* | GTA GGA GTA GAA GAA AAT GAG | (*6*) |
|  | TTA GTG GTG AGA AAA ACT AG |  |
| *srnA3* | GAA AGA AAT TTA CGG AGT AAA ATT ATG | This study |
|  | CCT TTT GTT TTA TCT AAC AAT GAA CA |  |
| *srnA4* | CTC CTG TCA AAG AAT AGA GGT GG | This study |
|  | CCA AGA GTT GAA TGG TAA ATC TGC |  |
| *srnM* | ACC CGA GAA CTA TCG AGC AT | (*6*) |
|  | TAC GGA CGC TTT TAC CTG GG |  |
| *srnT* | TGG CAT TGT CCC ACA AGA CA | (*6*) |
|  | CGT TGC CCT CCT GAC AGA TT |  |
| *Detection of S. salivarius adhesin genes* | | |
| *SrpA* (K10) | CAG ACT CGA TAA CCT GAC TAC C | This study |
|  | CTT AAC CTA GCG ACT GGT GAG |  |
| *CshA* | GGA GGA TTA CCA AAG TGG GT | This study |
|  | AGC GTC CCC TTC ATC AAT TG |  |
| *Adhesin-P1* | AAT AAC ACG AAA GGA CAT GGC | This study |
|  | GTG GGT GGT ATC TTA GCA TTC T |  |
| *Detection and quantification of oral bacteria* | | |
| *S. salivarius* | GTG TTG CCA CAT CTT CAC TCG CTT CGG | (*7*) |
|  | CGT TGA TGT GCT TGA AAG GGC ACC ATT |  |
| *P. gingivalis* | TGG TTT CAT GCA GCT TCT TT | (*8*) |
|  | TCG GCA CCT TCG TAA TTC TT |  |
| *T. forsythia* | GGG TGA GTA ACG CGT ATG TAA CCT | (*9*) |
|  | ACC CAT CCG CAA CCA ATA AA |  |
| *T. denticola* | CCT TGA ACA AAA ACC GGA AA | (*8*) |
|  | GGG AAA AGC AGG AAG CAT AA |  |
| *P. micra* | AAA CGA CGA TTA ATA CCA CAT GAG AC | (*10*) |
|  | ACT GCT GCC TCC CGT AGG A |  |
| *F. nucleatum* | AAG CGC GTC TAG GTG GTT ATG T | (*11*) |
|  | TGT AGT TCC GCT TAC CTC TCC AG |  |
| *16S rRNA* | TCC TAC GGG AGG CAG CAG T | (*12*) |
|  | GGA CTA CCA GGG TAT CTA ATC CTG TT |  |

**Table S6.** *In silico* Primer-BLAST predicted amplification targets for the *srnA2* qPCR assay

| **Species** | **Accession** | **Product Length**  **(bp)** | **Forward Binding**  **(nt)** | **Reverse Binding**  **(nt)** | **Mismatches**  **(strand)** |
| --- | --- | --- | --- | --- | --- |
| *S. salivarius* | CP090008.1 | 215 | 90558–90578 | 90364–90383 | 0 |
| *S. salivarius* | PQ082690.1 | 215 | 1–21 | 196–215 | 0 |
| *S. salivarius* | KT032116.1 | 215 | 8733–8753 | 8928–8947 | 0 |
| *S. salivarius* | CP040803.1 | 215 | 169262–169282 | 169068–169087 | 0 |
| *S. salivarius* | CP040803.1 | 215 | 5246-5266 | 5052-5071 | 0 |
| *S. pneumoniae* | KT630265.1 | 215 | 1648–1668 | 1843–1862 | 0 |
| *S. pneumoniae* | LR216040.1 | 215 | 1247646-1247666 | 1247452-1247471 | 0 |
| *S. pneumoniae* | CP031248.1 | 215 | 390998-391018 | 390804-390823 | 0 |
| *S. pneumoniae* | MF990783.1 | 215 | 325–345 | 520–539 | 1 (Rev) |
| *S. pneumoniae* | CP028436.1 | 215 | 1099625–1099645 | 1099820–1099839 | 1 (Rev) |
| *S. pneumoniae* | CP155538.1 | 215 | 972917–972937 | 973112–973131 | 1 (Rev) |
| *S. pneumoniae* | KT630266.1 | 215 | 1650–1670 | 1845–1864 | 1 (Rev) |
| *S. pneumoniae* | CP113267.1 | 215 | 930972–930992 | 931167–931186 | 1 (Rev) |
| *S. pneumoniae* | LK020704.1 | 215 | 17987–18007 | 18182–18201 | 1 (Rev) |
| *S. pneumoniae* | CP035256.1 | 215 | 1112646–1112666 | 1112841–1112860 | 1 (Rev) |
| *S. pneumoniae* | CP035255.1 | 215 | 1083683–1083703 | 1083878–1083897 | 1 (Rev) |
| *S. pneumoniae* | KC488257.1 | 215 | 22608–22628 | 22803–22822 | 1 (Rev) |
| *S. pneumoniae* | LS483523.1 | 215 | 1539406–1539426 | 1539212–1539231 | 1 (Rev) |
| *S. pneumoniae* | AP026924.1 | 215 | 1047470–1047490 | 1047276–1047295 | 1 (Rev) |

*srnA2* primers are listed in Table S5.

**References**

1. R. McNab *et al.*, Cell Wall-Anchored CshA Polypeptide (259 Kilodaltons) in Streptococcus gordonii Forms Surface Fibrils That Confer Hydrophobic and Adhesive Properties. **181**, 3087–3095 (1999).

2. C. R. Back *et al.*, The streptococcal multidomain fibrillar adhesin CshA has an elongated polymeric architecture. *Journal of Biological Chemistry* **295**, 6689–6699 (2020).

3. B. A. Bensing, R. Seepersaud, Y. T. Yen, P. M. Sullam, Selective transport by SecA2: an expanding family of customized motor proteins. *Biochim Biophys Acta* **1843**, 1674–1686 (2014).

4. G. Hajishengallis, T. Koga, M. W. Russell, Affinity and Specificity of the Interactions between Streptococcus mutans Antigen I/II and Salivary Components. **73**, 1493–1502 (1994).

5. K. P. Heim *et al.*, An intramolecular lock facilitates folding and stabilizes the tertiary structure of Streptococcus mutans adhesin P1. **111**, 15746–15751 (2014).

6. A. Barbour *et al.*, Discovery of phosphorylated lantibiotics with proimmune activity that regulate the oral microbiome. *Proceedings of the National Academy of Sciences* **120**, e2219392120 (2023).

7. T. Hoshino *et al.*, PCR detection and identification of oral streptococci in saliva samples using GTF genes. *Diagnostic Microbiology and Infectious Disease* **48**, 195–199 (2004).

8. K. Hyvärinen *et al.*, Detection and quantification of five major periodontal pathogens by single copy gene-based real-time PCR. *Innate Immunity* **15**, 195–204 (2009).

9. S. B. Ferreira *et al.*, An Interleukin-1β (IL-1β) Single-Nucleotide Polymorphism at Position 3954 and Red Complex Periodontopathogens Independently and Additively Modulate the Levels of IL-1β in Diseased Periodontal Tissues. *Infection and Immunity* **76**, 3725–3734 (2008).

10. C. Nonnenmacher, A. Dalpke, R. Mutters, K. Heeg, Quantitative detection of periodontopathogens by real-time PCR. *Journal of Microbiological Methods* **59**, 117–125 (2004).

11. M. Zepeda-Rivera *et al.*, A distinct Fusobacterium nucleatum clade dominates the colorectal cancer niche. *Nature* **628**, 424–432 (2024).

12. M. A. Nadkarni, F. E. Martin, N. A. Jacques, N. Hunter, Determination of bacterial load by real-time PCR using a broad-range (universal) probe and primers set. *Microbiology* **148**, 257–266 (2002).
